# Intranasal immunization with avian paramyxovirus type 3 expressing SARS-CoV-2 spike protein protects hamsters against SARS-CoV-2

**DOI:** 10.1038/s41541-022-00493-x

**Published:** 2022-06-28

**Authors:** Hong-Su Park, Yumiko Matsuoka, Cindy Luongo, Lijuan Yang, Celia Santos, Xueqiao Liu, Laura R. H. Ahlers, Ian N. Moore, Sharmin Afroz, Reed F. Johnson, Bernard A. P. Lafont, David W. Dorward, Elizabeth R. Fischer, Craig Martens, Siba K. Samal, Shirin Munir, Ursula J. Buchholz, Cyril Le Nouën

**Affiliations:** 1 RNA Viruses Section, Laboratory of Infectious Diseases, National Institute of Allergy and Infectious Diseases, National Institutes of Health, Bethesda, MD 20892 USA; 2 Infectious Disease Pathogenesis Section, Comparative Medicine Branch, National Institute of Allergy and Infectious Diseases, National Institutes of Health, Bethesda, MD 20892 USA; 3 SARS-CoV-2 Virology Core, Laboratory of Viral Diseases, National Institute of Allergy and Infectious Diseases, National Institutes of Health, Bethesda, MD 20892 USA; 4 Research Technologies Branch, Rocky Mountain Laboratories, National Institute of Allergy and Infectious Diseases, National Institutes of Health, Hamilton, MT 59840 USA; 5 Virginia-Maryland College of Veterinary Medicine, University of Maryland, College Park, MD 20742 USA

**Keywords:** Live attenuated vaccines, Viral vectors

## Abstract

Current vaccines against severe acute respiratory syndrome coronavirus 2 (SARS-CoV-2) are administered parenterally and appear to be more protective in the lower versus the upper respiratory tract. Vaccines are needed that directly stimulate immunity in the respiratory tract, as well as systemic immunity. We used avian paramyxovirus type 3 (APMV3) as an intranasal vaccine vector to express the SARS-CoV-2 spike (S) protein. A lack of pre-existing immunity in humans and attenuation by host-range restriction make APMV3 a vector of interest. The SARS-CoV-2 S protein was stabilized in its prefusion conformation by six proline substitutions (S-6P) rather than the two that are used in most vaccine candidates, providing increased stability. APMV3 expressing S-6P (APMV3/S-6P) replicated to high titers in embryonated chicken eggs and was genetically stable, whereas APMV3 expressing non-stabilized S or S-2P were unstable. In hamsters, a single intranasal dose of APMV3/S-6P induced strong serum IgG and IgA responses to the S protein and its receptor-binding domain, and strong serum neutralizing antibody responses to SARS-CoV-2 isolate WA1/2020 (lineage A). Sera from APMV3/S-6P-immunized hamsters also efficiently neutralized Alpha and Beta variants of concern. Immunized hamsters challenged with WA1/2020 did not exhibit the weight loss and lung inflammation observed in empty-vector-immunized controls; SARS-CoV-2 replication in the upper and lower respiratory tract of immunized animals was low or undetectable compared to the substantial replication in controls. Thus, a single intranasal dose of APMV3/S-6P was highly immunogenic and protective against SARS-CoV-2 challenge, suggesting that APMV3/S-6P is suitable for clinical development.

## Introduction

Severe acute respiratory syndrome coronavirus 2 (SARS-CoV-2) is a positive-sense, non-segmented, single-stranded RNA virus that is closely related to SARS-CoV-1 and Middle East respiratory syndrome coronavirus (MERS-CoV), all of which belong to the genus *Betacoronavirus* of the family *Coronaviridae*. SARS-CoV-2 emerged in 2019 as the causative agent of coronavirus disease 2019 (COVID-19) and created a pandemic and global public health crisis. Vaccines based on mRNA and replication-incompetent adenoviruses have been developed and have been effective in reducing the morbidity and mortality due to COVID-19^1–3^. However, many parts of the world have not yet had access to effective COVID-19 vaccines, and there is a continuing, urgent need for vaccines and therapeutics.

The SARS-CoV-2 envelope contains the spike (S) protein that, through its receptor-binding domain (RBD), binds to the host cellular receptor, angiotensin-converting enzyme 2 (ACE2)^4,5^ and mediates virus-cell membrane fusion required for viral entry. It is the primary target for neutralizing antibodies and also a target for T cell responses^6,7^. Newly synthesized S protein is present in a prefusion conformation that has been shown to be more immunogenic^8,9^. The stability of the prefusion conformation of the coronavirus S protein can be increased by genetic engineering, resulting in improved immunogenicity, as exemplified by SARS-CoV-1 and MERS-CoV^10^. The first generation of SARS-CoV-2 S protein stabilized in its prefusion form contained two proline substitutions at amino acids (aa) 986 and 987 (S-2P)^11^. A second generation of prefusion-stabilized S protein contained four additional proline substitutions at aa positions 817, 892, 899, and 942 [S HexaPro or S-6P^12^]. The S-6P version exhibited increased expression and stability compared to S-2P when expressed in mammalian cells while retaining its antigenicity^11,12^. The SARS-CoV-2 vaccine platforms that have received emergency use authorization (EUA), or those in development, are based mostly on the S-2P form^13–15^.

While the COVID-19 vaccines presently in human use have been remarkably effective in reducing COVID-19 morbidity and mortality, there is increasing recognition of breakthrough infections in vaccinated individuals^16,17^ as well as general concerns about the longevity of protection. Breakthrough infections have been associated with reduced titers of serum SARS-CoV-2-neutralizing antibodies^17^, which are both a protective effector and a marker for immunity in general. Breakthrough infection does not necessarily involve emerging variants with increased infectivity because infection with older variants is also being observed. However, although severe cases do occur, these usually are much less severe than those in virus-naive individuals, and appear to primarily involve the upper respiratory tract. Nonetheless, there is frequently shedding of high titers of virus which would promote spread. In addition, asymptomatic breakthrough infections – and shedding – likely occur that are not identified and facilitate spread. Current COVID-19 vaccines are administered parenterally and thus do not directly stimulate respiratory tract immunity. Therefore, it is important to evaluate additional vaccine approaches, in particular those involving direct immunization of the respiratory tract. In the present study, we developed a replication-competent, attenuated, intranasal, SARS-CoV-2 vaccine candidate based on avian paramyxovirus (APMV) serotype 3 vector.

APMVs are classified in the Order *Mononegavirales*, Family *Paramyxoviridae*, and are non-segmented, negative-sense, single-stranded RNA viruses that replicate in the cytoplasm and acquire a lipid envelope by budding through the plasma membrane. APMVs can be isolated from domestic and wild birds, with 21 serotypes identified to date^18^. APMV type 1, better known as Newcastle disease virus (NDV), is the most common and best-characterized of the APMVs and exemplifies their general properties. NDV infection of humans is rare and is largely restricted to bird handlers, is highly restricted due to host incompatibility, and causes mild or no illness. NDV is antigenically distinct from human pathogens^19^. Thus, humans generally lack pre-existing immunity to NDV that might otherwise interfere with vector infection and immunogenicity^20^. In an experimental setting, NDV and other APMVs can infect rodents and non-human primates by the respiratory route and cause a low level of replication that is restricted to the respiratory tract^21,22^. Additionally, NDV has also been used in humans as an oncolytic or cancer immunotherapy agent, usually in high doses given parenterally, and generally has been well-tolerated^23,24^. Recombinant NDV has also been used as an experimental vaccine vector to express viral antigens from pathogens including human parainfluenza virus type 3, respiratory syncytial virus, highly pathogenic influenza virus, SARS-CoV-1 and SARS-CoV-2^19,21,25–27^. However, NDV is a worldwide poultry pathogen that can cause serious outbreaks, and the NDV strains that have been the most promising as vectors have limited use due to their classification as select agents by the United States Department of Agriculture (USDA).

Based on the evaluation of several APMV serotypes, we recently identified APMV3 (strain Parakeet/Netherlands/449/75) as a promising alternative to NDV. In hamsters, APMV3 was infectious by the intranasal route, and replication was restricted to the respiratory tract^28^. This APMV3 strain does not appear to be a significant natural pathogen of poultry^29^, and in experimental infections was only mildly pathogenic in poultry^30^. This strain has also been characterized as low-pathogenic based on standard assays in embryonated chicken eggs and 1-day-old and 2-week-old chicks and turkeys^30^. A reverse genetics system was previously developed and the P/M intergenic region was identified as an optimal insertion site for foreign genes^31,32^. APMV3 has been evaluated in animal models as an experimental vaccine vector expressing the Ebola virus glycoprotein GP^31^ and the hemagglutinin HA protein of highly pathogenic avian influenza virus H5N1^33^. In the present study, we used APMV3 to express the SARS-CoV-2 S protein, stabilized in its prefusion form, and evaluated the immunogenicity and protective efficacy of a single intranasal dose against SARS-CoV-2 challenge in hamsters.

## Results

### Generation of APMV3 expressing the SARS-CoV-2 S protein

**Fig. 1:**
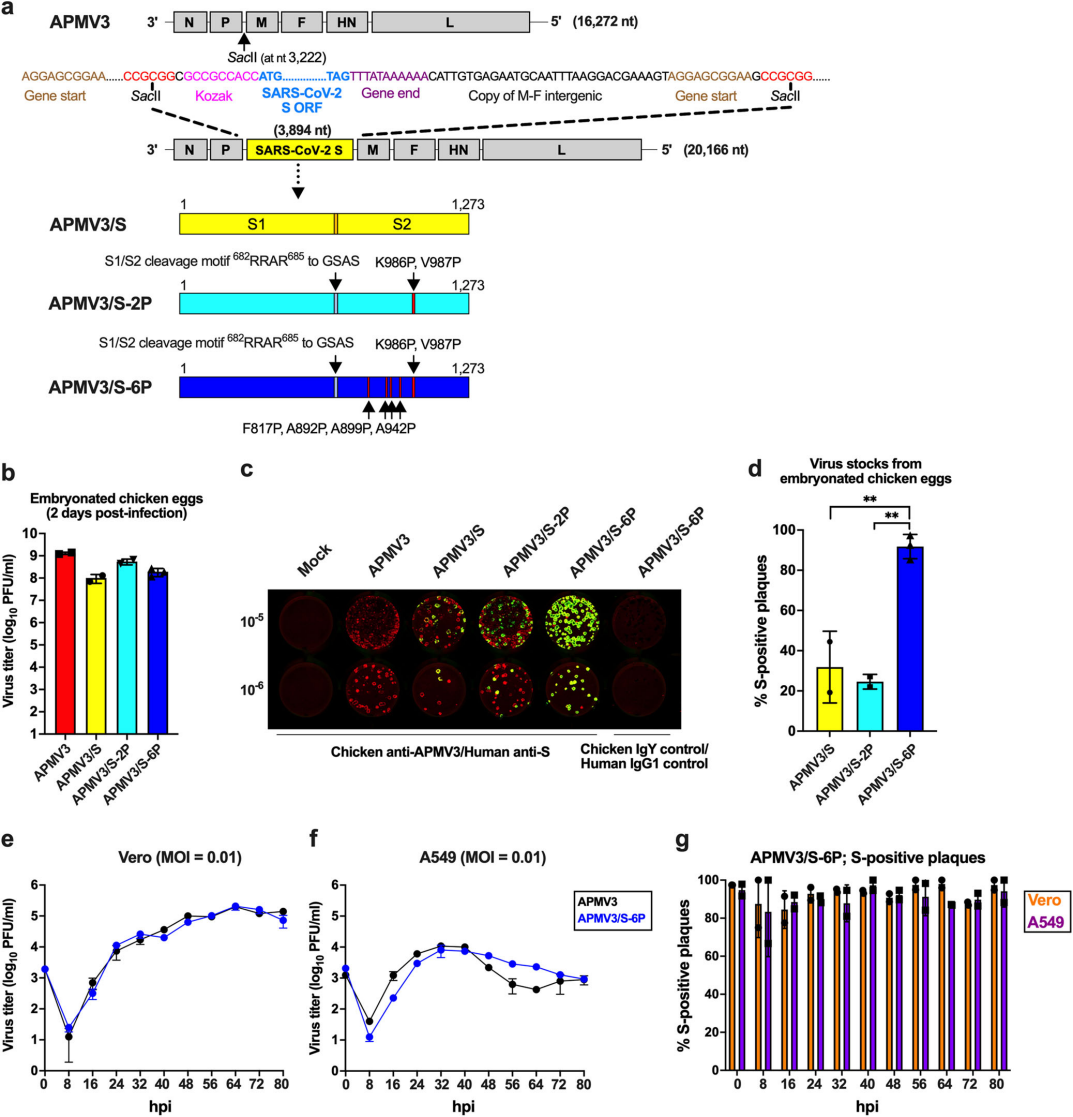
Generation and replication of APMV3 expressing a genetically stabilized prefusion form of SARS-CoV-2 S protein (APMV3/S-6P). **a** Diagram of the APMV3 genome and the SARS-CoV-2 S gene and encoded protein. A copy of the full-length SARS-CoV-2 S ORF (aa 1–1,273) from the WA1/2020 isolate of lineage A (GenBank MN908947) was codon-optimized for human expression, flanked by adapter sequence, and inserted between the APMV3 P and M genes under the control of a set of APMV3 gene-start and gene-end transcription signals for expression as a separate mRNA, and with a “Kozak” sequence for optimal initiation of translation (see Methods). Two additional versions were generated, expressing two different prefusion-stabilized versions of the S protein (APMV3/S-2P and APMV3/S-6P, see Methods and Results). Both prefusion-stabilized versions of the S ORF include RRAR-to-GSAS changes that ablate the S1/S2 cleavage site. The presence of two (APMV3/S-2P) or six (APMV3/S-6P) stabilizing proline substitutions is indicated by red lines marked by arrows. **b** Virus titers of APMV3 and derivatives in the allantoic fluid of embryonated chicken eggs on day 2 post-infection determined by plaque assay on Vero cells as described in Methods. Error bars indicate the standard deviation (SD) of the mean from two (APMV3, APMV3/S and APMV3/S-2P) or three (APMV3/S-6P) independent virus stocks. **c** Stability of expression of the different versions of SARS-CoV-2 S protein. Vero cells were inoculated with ten-fold serial dilutions of egg-grown stocks of APMV3 and derivatives, incubated under methylcellulose overlay, fixed on day 4 post-infection, and incubated with a chicken anti-APMV3 antibody and a human anti-SARS-CoV-2 S monoclonal antibody CR3022, followed by incubation with infrared dye-labeled secondary antibodies. Infrared images were obtained, and APMV3 and SARS-CoV-2 S immunostainings were pseudocolored red and green, respectively. Plaques expressing both APMV3 and SARS-CoV-2 S antigens appear yellow whereas plaques expressing only APMV3 appear red. Chicken IgY and human IgG1 antibodies were used as negative controls. **d** Percentage of S-positive plaques out of total plaques in immunoplaque assays of egg-grown stocks of APMV3/S, APMV3/S-2P, and APMV3/S-6P. Error bars indicate SD of the means from two (APMV3 and APMV3/S) or three (APMV3/S-2P and APMV3/S-6P) independent virus stocks. **p ≤ 0.01, one-way ANOVA with Tukey post-hoc test. **e**, **f** Multicycle growth kinetics of APMV3 and APMV3/S-6P in Vero (**e**) and A549 cells (**f**). Replicate sub-confluent monolayers were infected in quadruplicate with each virus using an MOI of 0.01 PFU/cell. After 2 h incubation, Vero cells were washed once and replenished with Opti-MEM containing 1% l-glutamine and 2% TrypLE Select. A549 cells were washed and replenished with F-12K containing 1% l-glutamine and 0.5% TrypLE Select. Every 8 h from 8 to 80 hpi, cells from duplicate wells per virus and time point were harvested by scraping into media, subjected to short centrifugation to collect the clarified supernatant that was aliquoted and snap-frozen followed by storage at −80 °C. Virus titers were determined in duplicate by immunoplaque assay on Vero cells at 37 °C as described in Methods. The third replica wells from the growth curves were used to evaluate the expression of SARS-CoV-2 S and APMV3 N proteins by Western blotting, and the fourth wells were used to analyze the expression of SARS-CoV-2 S protein by flow cytometry (see Fig. 2 and S2). **g** Percentage of S-positive plaques out of total plaques generated by APMV3/S-6P in Vero (circles) and A549 cells (squares) during titrations of clarified supernatants from the multicycle growth kinetics performed at an MOI of 0.01 as described in (**e**) and (**f**). Error bars indicate SD of the mean.

We used APMV3 to express the SARS-CoV-2 S protein, the main protective antigen of SARS-CoV-2. The open reading frame (ORF) encoding the full-length SARS-CoV-2 S protein (1,273 aa) of the first available sequence [GenBank MN908947;^34^, assigned to lineage B^35^] was codon-optimized for human expression. Three versions of the ORF were made encoding: (i) the unmodified full-length wild-type (WT) S protein (Fig. 1a, top construct); (ii) S protein stabilized in prefusion conformation by two proline substitutions at aa positions 986 and 987 (S-2P)^11^ (Fig. 1a, second from bottom); and (iii) S protein stabilized in the prefusion conformation by the above-mentioned two proline substitutions at aa positions 986 and 987 plus four additional proline mutations at aa positions 817, 892, 899, and 942 (S-6P), which were shown to confer increased stability to the prefusion conformation^12^ (Fig. 1a, bottom). In addition, in the S-2P and S-6P versions, the S1/S2 polybasic furin cleavage motif “RRAR” was ablated by aa substitutions (RRAR-to-GSAS)^11^.

Each of the three ORFs was designed to be framed by nucleotide adapters for insertion as an additional gene between the APMV3 P and M genes in the APMV3 genome (Fig. 1a, Methods). The design placed the S ORF under control of APMV3 gene-start and gene-end signals to direct transcription by the APMV3 polymerase complex into a separate mRNA. The three different versions of the S ORF were synthesized *de novo* and inserted into a cDNA clone encoding a full-length positive-sense copy of the APMV3 genome. These cDNAs were transfected with support plasmids into baby hamster kidney cells stably expressing T7 RNA polymerase (BSR T7/5^36^), resulting in the recovery, by reverse genetics, of the viruses APMV3/S, APMV3/S-2P, and APMV3/S-6P. In parallel, we recovered the parental APMV3-empty vector from cDNA.

Viruses recovered from BSR T7/5 cells were passaged once or twice on Vero cells, a suitable cell line for vaccine manufacture. APMV3 replication on Vero cells is dependent on the addition of trypsin or allantoic fluid to provide the protease needed for cleavage activation of the APMV3 F0 precursor^37^. In the present study, the addition of trypsin to the cell culture medium, as well as gentle manipulation of virus-infected Vero cells during the harvest by scraping and vortexing, significantly increased virus titers in clarified supernatants from the infected Vero cells (Supplementary Fig. 1). Nonetheless, the recovered APMV3s replicated only to moderate titers in Vero cells, with final titers of virus stocks not exceeding 5.5 log_10_ plaque-forming units (PFU) per mL (Supplementary Fig. 1).

To generate high-titer virus stocks, we further amplified the recombinant APMV3s by inoculating Vero-grown material into the allantoic cavity of 11-day-old embryonated chicken eggs, which are another suitable substrate for vaccine manufacture. Two days after inoculation, allantoic fluids were harvested. The empty APMV3 vector replicated to high titers, reaching a mean titer of 9.1 log_10_ PFU/ml on day 2 post-infection (from *n* = 2 independent recoveries, Fig. 1b). APMV3/S (*n* = 2), APMV3/S-2P (*n* = 2), and APMV3/S-6P (*n* = 3) also replicated efficiently in embryonated chicken eggs, reaching mean titers of 8 log_10_, 8.7 log_10_, and 8.3 log_10_ PFU/ml, respectively (Fig. 1b).

Next, we evaluated the stability of the S expression by Vero- and egg-derived APMV3 by a dual-staining immunoplaque assay. This assay was designed to detect co-expression of S and APMV3 proteins by individual plaques that had developed on Vero cell monolayers under a methylcellulose overlay. The monolayers were fixed and immunostained using a chicken hyperimmune serum to APMV3 and a human monoclonal antibody to the S protein. Following staining with species-specific secondary antibodies conjugated to infrared fluorescent dyes, infrared imaging revealed the presence of APMV3 proteins and S protein (pseudocolored in red and green, respectively): plaques expressing APMV3 proteins but not S would appear red and plaques co-expressing APMV3 protein and S protein would appear yellow (Fig. 1c). No significant differences in plaque morphology were observed between the empty APMV3 vector and APMV3 expressing any of the three versions of S protein (Fig. 1c). The expression of the three different versions of S by APMV3 vectors grown in Vero cells was stable, with dual-staining for both APMV3 and S protein detectable in >99% of plaques (not shown).

However, after amplification in embryonated chicken eggs, approximately 92% (mean from three independent virus recoveries) of APMV3/S-6P plaques were positive for S immunostaining, whereas only 32% (mean from two independent recoveries) and 25% (mean from two independent recoveries) of the APMV3/S and APMV3/S-2P plaques were positive for S immunostaining, respectively (Fig. 1d). Thus, substantial fractions of APMV3/S and APMV3/S-2P virions had lost the ability to express the S protein during propagation in chicken eggs. Sanger sequencing of the S ORF of two egg-grown APMV3/S stocks and one of the two egg-grown APMV3/S-2P stocks did not reveal any nonsense mutations. The second egg-grown APMV3/S-2P stock was generated by pooling allantoic fluid from 19 individual infected eggs. Prior to pooling, aliquots from six of the 19 eggs were used to extract viral RNA and sequence the S ORF by Sanger sequencing. No mutation in the S ORF was identified in the viruses harvested from three of these six eggs. However, nonsense mutations resulting in stop codons were identified at codon 129 (virus harvested from two eggs) or codon 291 of the S ORF (virus from one egg). We also performed complete-genome sequencing of one egg-grown APMV3 stock and three egg-grown APMV3/S-6P stocks, and did not detect any mutations. Thus, the introduction of the six proline substitutions provided increased stability of expression of S from the APMV3 vector during replication in embryonated chicken eggs. Due to their low yields in Vero cells and genetic instability in embryonated chicken eggs, APMV3/S and APMV3/S-2P were not evaluated further, while APMV3/S-6P was further investigated in vitro and in the hamster model.

### In vitro characterization of APMV3/S-6P

To compare the multicycle replication of APMV3/S-6P and APMV3, we infected Vero or human lung A549 cells with the egg-grown virus stocks at a low multiplicity of infection (MOI) of 0.01 at 37 °C in the presence of trypsin. In Vero cells, both viruses replicated with similar kinetics and reached peak titers of around 5.3 log_10_ PFU/ml at 64 hours post-infection (hpi, Fig. 1e). APMV3/S-6P and APMV3 also replicated in A549 cells, albeit less efficiently than in Vero cells, reaching peak titers at 32 hpi of only 3.9 log_10_ and 4.0 log_10_ PFU/ml, respectively (Fig. 1f).

To evaluate if S was stably expressed by APMV3/S-6P in Vero and A549 cells, we determined the percentage of plaques that expressed S over the multicycle replication experiments by the dual-staining immunoplaque assay (Fig. 1g). Following passage in Vero or A549 cells, 83 to 98% of APMV3/S-6P plaques were S-positive, with the percentages being very similar between the two cell lines. In addition, the percentage of S-expressing plaques did not decrease over the time course, suggesting that S expression by APMV3/S-6P was stable in both cell lines.

**Fig. 2:**
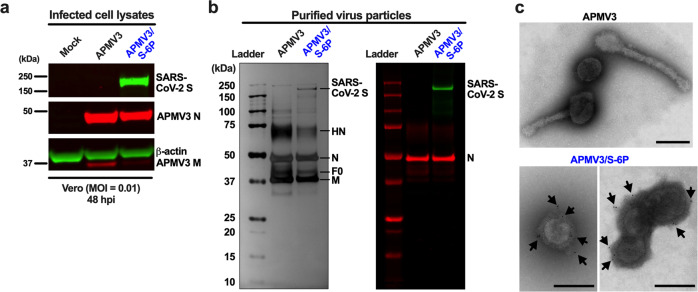
Expression of virus proteins and incorporation of the SARS-CoV-2 S protein into the vector particles. **a** Viral protein expression in Vero cells. From the experiment described in Figs. 1e, f, one additional replicate well of Vero cells per virus, which had been infected with an MOI of 0.01 PFU/cell of the indicated virus, was harvested at 48 hpi, and cell lysates were prepared and analyzed by SDS-PAGE under reducing and denaturing conditions. The APMV3 N and SARS-CoV-2 S protein bands were visualized by Western blotting using primary rabbit anti-APMV3 serum and goat anti-SARS-CoV-2 S serum, followed by incubation with IRDye-conjugated secondary antibodies and infrared imaging (see Methods). A mouse anti-β-actin antibody was included to provide a loading control. Panels were derived from the same gel (shown in supplemental Fig. 5). **b**, **c** Incorporation of the SARS-CoV-2 S-6P protein into the vector particles. **b** Preparations of APMV3 and APMV3/S-6P were sucrose-purified, and three μg of protein from each preparation were analyzed per lane by SDS-PAGE under reducing and denaturing conditions. A gel was subjected to silver staining (left panel). In parallel, another gel was analyzed by Western blotting to detect the presence of SARS-CoV-2 S along with APMV3 N (right panel). Images are representative of three independent experiments. The identities of the bands annotated in the silver-stained gel were confirmed by proteomics analysis (see Results). Ladder, molecular size marker. **c** Incorporation of SARS-CoV-2 S in APMV3/S-6P virus particles was also evaluated by immunogold staining and electron microscopy. APMV3/S-6P and APMV3 virus preparations were incubated with goat anti-SARS-CoV-2 S serum, followed by sucrose purification. Then, sucrose-purified viruses were fixed and probed with gold-labeled secondary antibody before analysis by electron microscopy. A representative image of three pleomorphic APMV3 particles is shown on the top panel. Representative images of APMV3/S-6P are shown in the bottom panels (one and three virus particles in the left and right panels for APMV3/S-6P, respectively). Gold particles are indicated by black arrows. Scale bars indicate 200 nm.

We used additional wells from the multicycle replication experiment described in Figs. 1e, f to characterize the expression of viral proteins in Vero and A549 cells by Western blotting and flow cytometry (Supplementary Fig. 2). Cell lysates were prepared and analyzed under reducing and denaturing conditions by SDS-polyacrylamide gel electrophoresis (PAGE) and Western blotting using antisera against APMV3 and SARS-CoV-2 S. A representative Western blot of Vero cells harvested 48 hpi is shown in Fig. 2a. In lysates of APMV3- and APMV3/S-6P-infected Vero cells, we detected a strong band consistent in size with APMV3 N. The APMV3 N protein accumulated similarly in APMV3- and APMV3/S-6P-infected Vero cells from 24 to 80 hpi (Supplementary Fig. 2a). In the APMV3/S-6P-infected cell lysate only, we additionally detected a single strong band consistent in size with the uncleaved SARS-CoV-2 S0 protein (Fig. 2a). The SARS-CoV-2 S protein also accumulated in APMV3/S-6P-infected Vero cells from 24 to 80 hpi (Supplementary Fig. 2b). Using replicate wells from the same experiment, we determined the percentage of S-expressing Vero cells over time by flow cytometry (Supplementary Fig. 2c; see supplementary Fig. 3 for the gating strategy). This showed that, at 64 hpi, about 15% of the Vero cells inoculated with APMV3/S-6P were expressing SARS-CoV-2 S (Supplementary Fig. 2c). This percentage declined from 64 to 80 hpi. As described above, about 98% of the plaques produced by APMV3/S-6P expressed S in Vero cells at 64 hpi (Fig. 1g). Thus, S was an appropriate marker to evaluate the rate of APMV3/S-6P-infected cells. In A549 cells, the maximum expression levels of APMV3 N and SARS-CoV-2 S were reached around 48 hpi and were five- and eight-fold lower than those at 80 hpi in Vero cells, respectively (Supplementary Fig. 2d, e).

In an additional set of three independent experiments, Vero and A549 cells were infected with APMV3 or APMV3/S-6P using higher MOIs of 1 or 10 PFU/cell (Supplementary Fig. 2f, g). Cells were harvested at 24 hpi, and the proportion of cells expressing SARS-CoV-2 S protein was analyzed by flow cytometry. About 40 and 80% of the live single permeabilized Vero cells were positive for S protein at 24 hpi with an MOI of 1 and 10 PFU/cell, respectively (Supplementary Fig. 2f), while only about 15 and 26% of A549 cells had the S protein detectable at an MOI of 1 and 10 PFU/cell, respectively (Supplementary Fig. 2g). Thus, A549 cells were susceptible to APMV3/S-6P infection, but their permissiveness seemed lower than that of Vero cells.

### Incorporation of SARS-CoV-2 S in APMV3/S-6P virus particles

To investigate whether SARS-CoV-2 S was incorporated in APMV3/S-6P virions, we purified APMV3 and APMV3/S-6P that had been grown to high titers in embryonated chicken eggs by ultracentrifugation through discontinuous sucrose gradients. To analyze the protein composition, virus preparations were subjected to SDS-PAGE followed by silver staining (Fig. 2b, left panel) or Western blotting (Fig. 2b, right panel). A band with a molecular size corresponding to that of SARS-CoV-2 S was apparent on the silver-stained gel in the APMV3/S-6P preparation, but not in the APMV3 preparation. Immunostaining confirmed the presence of SARS-CoV-2 S in sucrose-purified preparations of APMV3/S-6P (Fig. 2b, right panel). Silver staining revealed additional protein bands in both the APMV3 and APMV3/S-6P preparations (Fig. 2b, left panel). These protein bands were identified, by mass spectrometry of gel bands excised from a Coomassie blue-stained gel run in parallel, as APMV3 HN (estimated molecular weight: 70 kDa), N (45–50 kDa), F (40–45 kDa), and M (40 kDa). HN in particular was somewhat more abundant in the APMV3 preparation than in the APMV3/S-6P preparation. Western blotting confirmed that APMV3 N was present in similar amounts in both APMV3 and APMV3/S-6P preparations.

We also evaluated the incorporation of S protein into APMV3/S-6P particles by immuno-electron microscopy (Fig. 2c). We incubated egg-grown APMV3 and APMV3/S-6P with goat polyclonal antibodies raised against a recombinantly expressed secreted form of S-2P^38^, followed by purification by ultracentrifugation through a discontinuous sucrose gradient. Purified preparations were labeled with a secondary anti-goat antibody conjugated with gold particles, followed by negative staining. Electron microscopy revealed the presence of abundant pleomorphic particles surrounded by envelope glycoproteins, consistent with APMV3 virions. No obvious differences in morphology between WT APMV3 and APMV3/S-6P were observed. Immunogold labeling was associated with the glycoprotein layer surrounding the APMV3/S-6P particles (Fig. 2c, bottom panels), indicating the presence of S protein in the APMV3/S-6P envelope. No immunogold labeling was detected on APMV3 virus particles (Fig. 2c, top panel). This observation further confirmed that SARS-CoV-2 S was incorporated in the APMV3/S-6P particles.

### Replication and genetic stability of APMV3/S-6P in hamsters

APMV3 replicates to moderate levels (~3 log_10_ PFU/g lung tissue) in the respiratory tract of golden Syrian hamsters without any gross clinical disease^28^. The hamster ACE2 sequence shares a high similarity with human ACE2, and SARS-CoV-2 replicates to higher titers than APMV3 in hamsters (~7 log_10_ PFU/g lung tissue) and induces moderate clinical disease^39–41^. Thus, golden Syrian hamsters are an appropriate animal model to evaluate both the replication and immunogenicity of APMV3 vectors and protection against SARS-CoV-2 challenge.

**Fig. 3:**
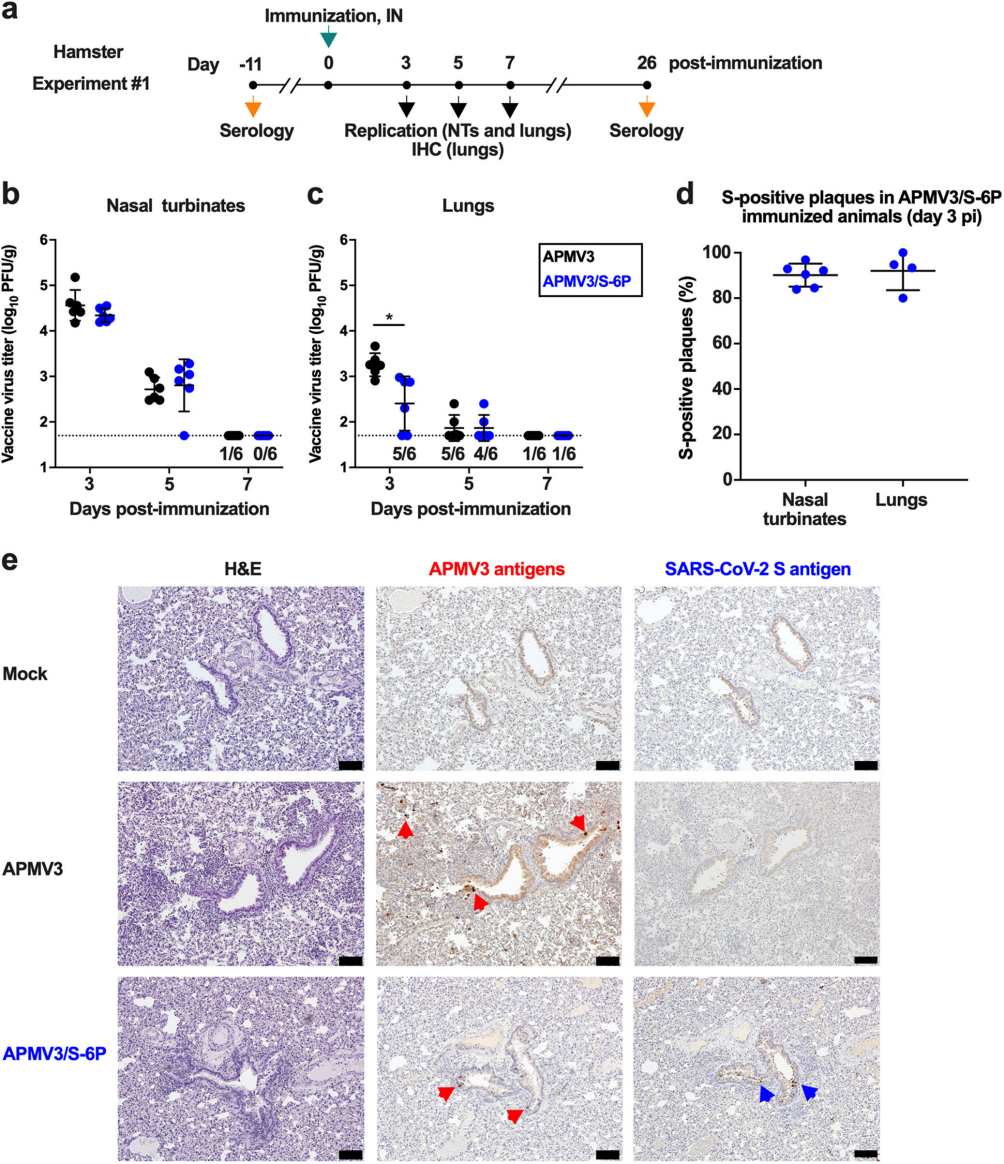
Replication of APMV3 and APMV3/S-6P in hamsters (from Experiment #1). **a** Timeline of immunization and sampling for Experiment #1. NTs, nasal turbinates; IHC, immunohistochemistry, IN, intranasal. Hamsters (*n* = 45 per group) were immunized intranasally with 6 log_10_ PFU of APMV3/S-6P or APMV3. On days 3, 5, and 7, six animals per group were sacrificed for virus titration (see panels **b**–**d**), and another two animals per group per day were sacrificed for IHC analysis (see panel **e**). Sera were collected from the remaining 21 animals per group on day 26 (prebleed sera had been taken on day -11) for analysis (see Supplementary Fig. 3). **b**, **c** Virus titers in NTs (**b**) and lungs (**c**) were determined by immunoplaque assay as described in Methods. The limit of detection, indicated by a dotted line, was 50 PFU per g of tissue. The numbers of animals with virus detectable at or above the limit of detection are indicated above the *x*-axis. The means with SD are shown. *p ≤ 0.05, two-way ANOVA with Sidak post-hoc test. **d** Stability of S expression on day 3 pi as determined by dual-staining immunoplaque assay. The means and SD of the percentages of S-positive plaques are shown. **e** IHC analysis of lung tissues. Serial sections were immunostained for APMV3 and SARS-CoV-2 S antigen using hyperimmune antisera against APMV3 virions and secreted S-2P protein, respectively. Representative images from day 5 pi are shown. Areas with bronchial epithelial cells positive for APMV3 and SARS-CoV-2 S are marked by red and blue arrowheads, respectively (×10 magnification; a 100 μm size bar is shown in the bottom right corners).

To evaluate virus replication and immunogenicity, we inoculated six-week-old hamsters in groups of 45 intranasally with a dose of 6 log_10_ PFU of APMV3/S-6P or the empty APMV3 vector per animal (Experiment #1, see Fig. 3a for the timeline). On days 3, 5, and 7 post-immunization (pi), six hamsters per group were euthanized, and virus replication was evaluated in the nasal turbinates (NTs) and lungs (Fig. 3b, c), as well as in brain tissue, kidneys, spleen, liver, intestines, and blood. We found that APMV3 and APMV3/S-6P replicated similarly well in the NTs reaching 4.5 log_10_ and 4.3 log_10_ PFU/g, respectively on day 3 pi (Fig. 3b). Titers of both viruses were lower by day 5 pi (2.7 log_10_ and 3.0 log_10_ PFU/g for APMV3 and APMV3/S-6P, respectively), and no replication was detected on day 7 in both groups, except for one animal in the APMV3 group in which replication in the NTs was detected at the limit of detection.

Both viruses replicated to lower titers in the lungs than in the NTs (Fig. 3c); the peak titers of APMV3 and APMV3/S-6P on day 3 pi reached 3.3 log_10_ PFU/g (70-fold lower compared to NTs) and 2.7 log_10_ PFU/g (20-fold lower compared to NTs), respectively. The mean peak titer of APMV3/S-6P was four-fold lower compared to the APMV3-empty-vector control, suggesting that the insertion of the S gene reduced APMV3 replication in the lungs. Lung titers of both viruses were lower by day 5. On day 7, only one animal from each group had virus detectable in the lungs, with titers at the limit of detection.

The stability of S expression by APMV3/S-6P was evaluated by dual-staining immunoplaque assay from animals necropsied on day 3 pi with virus detectable by titration in respiratory tissues (NTs and lungs from six and four hamsters, respectively; Fig. 3d). About 90 and 92% of the APMV3/S-6P plaques from the NTs and lungs, respectively, expressed S (Fig. 3d), suggesting that S expression by APMV3/S-6P was stable in hamsters. No replication of APMV3 and APMV3/S-6P was detected in the spleen, intestine, brain, kidney, liver, and blood, confirming that APMV3 replication was restricted to the respiratory tract and was not altered by the introduction of S in its prefusion form.

On days 3, 5, and 7, lung tissues were harvested from two additional animals per group and processed for immunohistochemistry (IHC) analysis to detect the expression of APMV3 and SARS-CoV-2 S antigen. Representative hematoxylin and eosin (H&E) staining and IHC images from day 5 are shown in Fig. 3e. We detected APMV3 and SARS-CoV-2 antigen (indicated by colored arrows) in columnar bronchial epithelial cells. Antigen-positive cells were largely limited to the bronchial and bronchiolar airway epithelium, only present in a few locations and only on day 5 pi.

### Immunogenicity in hamsters

**Fig. 4:**
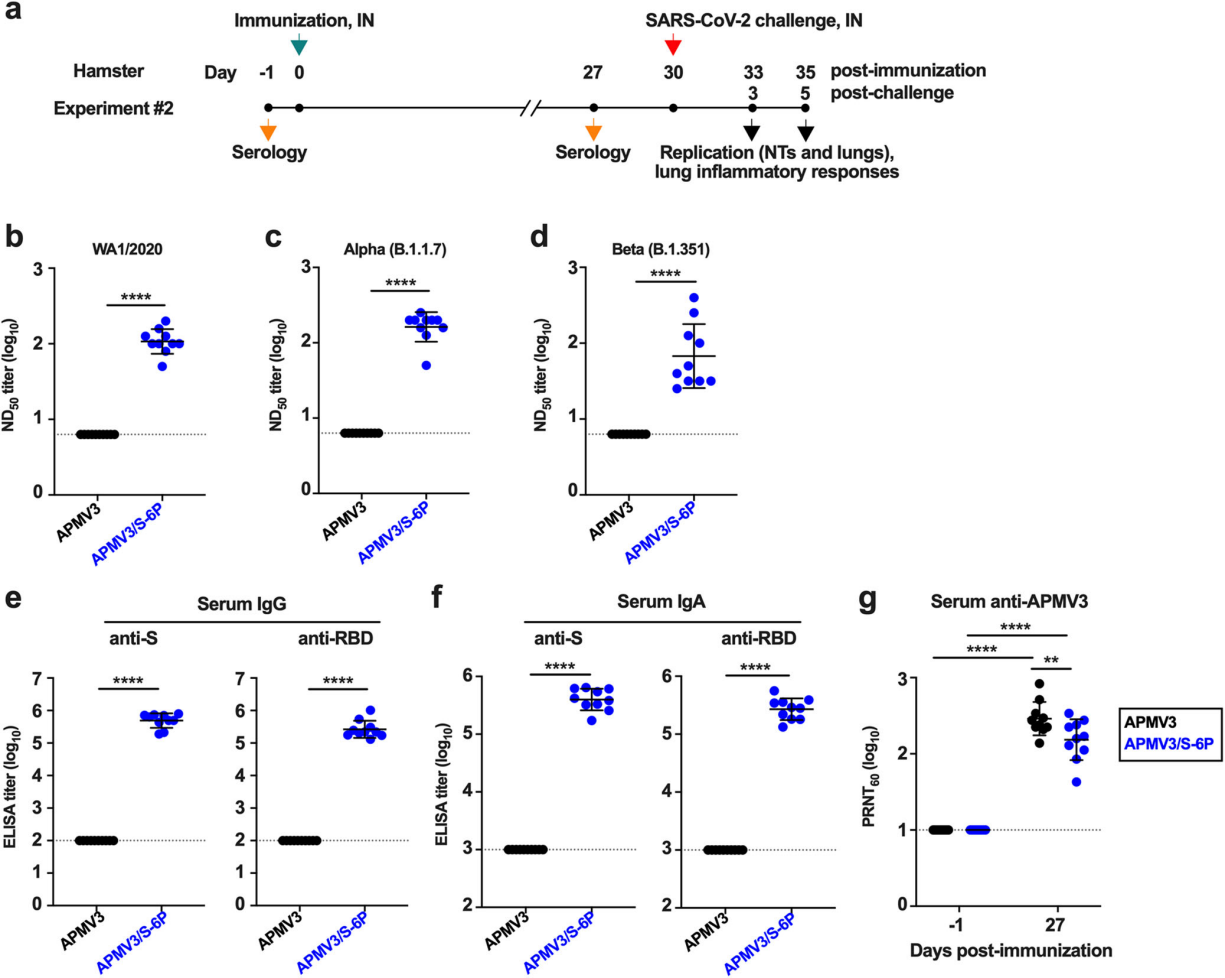
Immunogenicity of APMV3 and APMV3/S-6P in hamsters (from Experiment #2). **a** Timeline of immunization, sampling and challenge for Experiment #2. Groups of hamsters were immunized intranasally with 6 log_10_ PFU of APMV3/S-6P or APMV3. Sera were collected on days -1 and 27 pi. Analysis of the sera are shown in panels b-g; see Fig. 5 for the results of the SARS-CoV-2 challenge. **b**–**d** The SARS-CoV-2 ND_50_ were determined on Vero E6 cells against isolates WA1/2020 (lineage A) (**b**), USA/CA_CDC_5574/2020 (lineage B.1.1.7, Alpha variant) (**c**), and USA/MD-HP01542/2021 (lineage B.1.351, Beta variant) (**d**). ND_50_ titers are expressed in log_10_. The limit of detection, indicated by a dotted line, is 0.8 log_10_. **e**, **f** End-point ELISA titers expressed in log_10_ for serum IgG (**e**) and IgA (**f**) to a secreted prefusion-stabilized form (aa 1–1208) of the S protein (left panels) or to a fragment of the S protein (aa 328–531) containing SARS-CoV-2 receptor-binding domain (RBD) (right panels). The limit of detection was 2 and 3 log_10_ for IgG and IgA, respectively (dotted lines). **g** 60% plaque reduction neutralization titers (PRNT_60_) of the sera collected on days -1 and 27 pi against APMV3. The limit of detection was 1 log_10_ (dotted line). In each graph, the means and SD are shown. **p ≤ 0.01, ****p ≤ 0.0001, Mann-Whitney test (**b**–**f**) or two-way ANOVA with Sidak post-hoc test (**g**).

In a continuation of Experiment #1 described above (see Fig. 3a for the timeline), we collected sera on day 26 pi from the remaining 21 animals per group. In addition, we performed a second study (Experiment #2, see Fig. 4a for the timeline), also in six-week-old hamsters, in which two groups of 10 hamsters each were immunized intranasally with the same inocula as in Experiment #1 (6 log_10_ PFU of APMV3/S-6P or empty APMV3 vector). Sera were obtained on day 27 after immunization. Analyses of the sera from Experiments #1 and #2 are shown in Supplementary Fig. 4 and Fig. 4, respectively, and described below.

The SARS-CoV-2-neutralizing antibody titers were measured by 50% neutralizing dose (ND_50_) assays. In both experiments, hamsters immunized with APMV3/S-6P developed high levels of neutralizing antibodies against the homologous WA1/2020 isolate (mean ND_50_ titers of 2.5 and 2.0 log_10_ in Experiments #1 and #2, respectively; Supplementary Fig. 4a, b). In addition, using sera from Experiment #2 alone, we also evaluated the serum neutralizing titers against representatives of two variants being monitored, Alpha (lineage B.1.1.7) and Beta (lineage B.1.351). Serum antibodies from APMV3/S-6P-immunized hamsters efficiently neutralized live virus of the SARS-CoV-2 Alpha variant (mean ND_50_ titer of 2.2 log_10_, Fig. 4c), as well as of the Beta variant, although the titers against the latter variant were 1.6-fold lower (mean ND_50_ titer of 1.8 log_10_) and more variable among individual animals (ND_50_ titers ranging from 1.4 to 2.6 log_10_, Fig. 4d).

In both experiments, we also measured serum IgG titers against a recombinantly expressed secreted form of the SARS-CoV-2 S protein (aa 1–1,208; Supplementary Fig. 4b, e, left panel) and a recombinantly expressed fragment of the S protein bearing the RBD (aa 328–531; Supplementary Fig. 4c, e, right panel) by ELISA. In both studies, APMV3/S-6P induced a strong antibody response against the S protein (mean titers of 5.6 and 5.7 log_10_ in Experiment #1 and #2, respectively) and against the RBD (mean titer = 5.7 and 5.4 log_10_, in Experiment #1 and #2, respectively). The similarity in titers between the anti-S and anti-RBD responses suggested that most of the anti-S response was directed against the RBD. In addition, in Experiment #2, we evaluated serum IgA titers against S and the RBD by dissociation-enhanced lanthanide time-resolved fluorescent immunoassay (DELFIA-TRF) (Fig. 4f). This showed that APMV3/S-6P induced strong anti-S- and anti-RBD-specific IgA responses (mean titers of 5.6 and 5.4 log_10_, respectively). As expected, the SARS-CoV-2-neutralizing antibody titers and the anti-S and anti-RBD IgG ELISA titers of the sera collected from the APMV3-empty-vector-immunized groups in both experiments were below the limit of detection (Fig. 4b–f and Supplementary Fig. 4a–c).

Finally, in both experiments, there were strong serum neutralizing antibody responses against the APMV3 vector: in Experiment #1, the responses were not significantly different between the APMV3 and APMV3/S-6P groups (Supplementary Fig. 4d), whereas in Experiment #2 the response was modestly but significantly reduced in the APMV3/S-6P group (Fig. 4g).

### APMV3/S-6P protects hamsters from a SARS-CoV-2 challenge

**Fig. 5:**
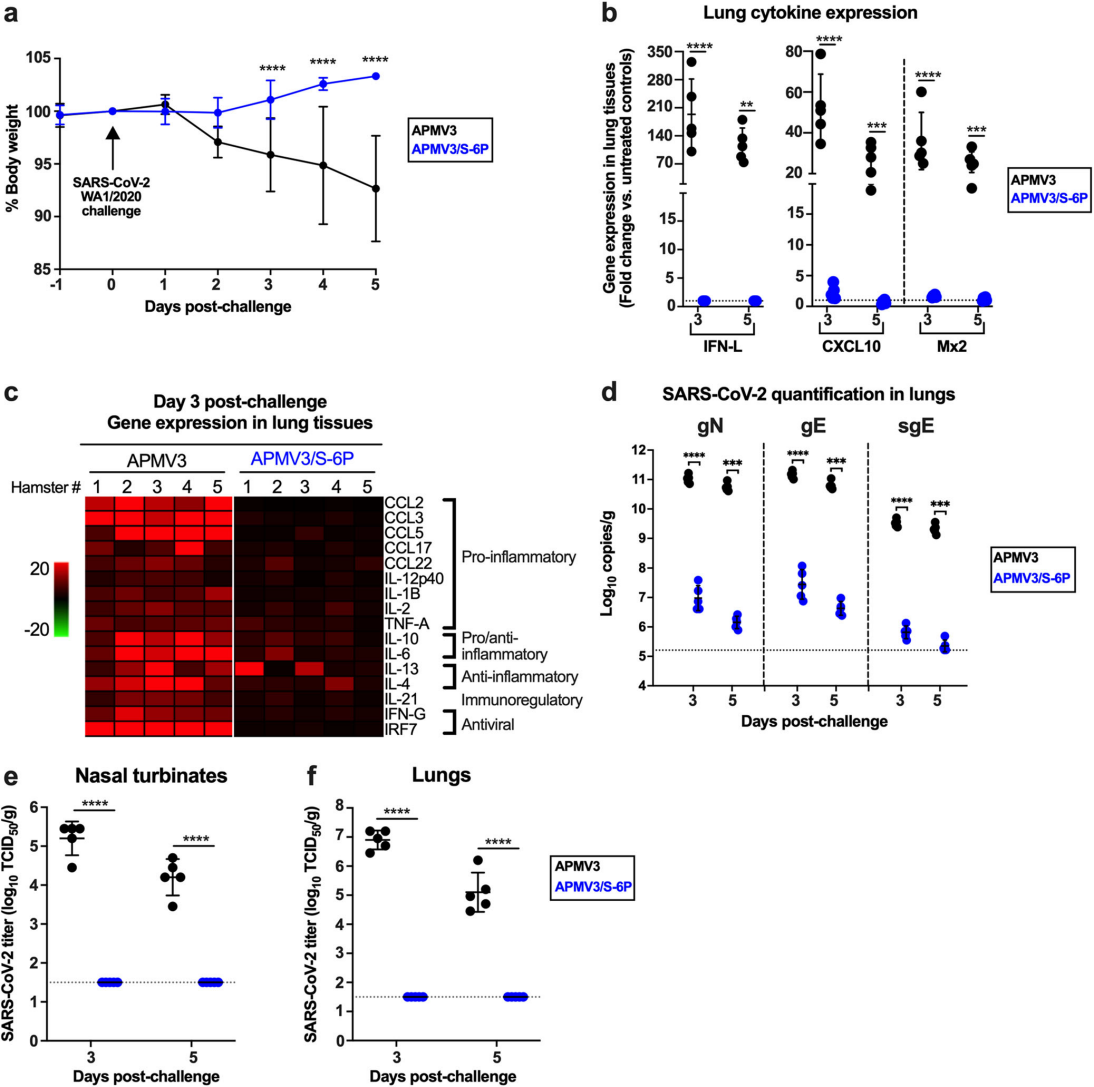
Protective efficacy of APMV3/S-6P against a SARS-CoV-2 challenge (from Experiment #2). As part of Experiment #2 (see Fig. 4a for the timeline) hamsters (*n* = 10 per group) immunized intranasally with 6 log_10_ PFU per animal of APMV3/S-6P or APMV3 were challenged intranasally on day 30 with 4.5 log_10_ TCID_50_ per animal of the SARS-CoV-2 WA1/2020. **a** Body weights were measured from day -1 to day 5 post-challenge and expressed as % body weight relative to the day 0 time point (*n* = 10 hamsters per group from day -1 to day 3 and *n* = 5 hamsters per group from day 4 to day 5). The means and SD are shown. ****p ≤ 0.0001, mixed effects analysis with Sidak post-hoc test. **b**–**f** Five hamsters per group were euthanized on days 3 and 5 post-challenge, and NTs and lungs were harvested, homogenized, and aliquots were snap-frozen until further processing. **b**–**c** Total RNA was extracted from lung homogenates and subjected to TaqMan RT-qPCR to evaluate the expression of selected genes related to inflammatory/antiviral responses. qPCR data were analyzed using the ΔΔCt method, normalized to β-actin, and expressed as fold-change over the average expression levels of three mock-immunized, mock-challenged hamsters. The three most up-regulated genes (IFN-L, CXCL10 and Mx2) are shown in (**b**) while the relative expression levels of the remaining 16 genes on day 3 after challenge are shown in (**c**) as a heat map (*n* = 5 per group). **p ≤ 0.01, ***p ≤ 0.001 and ****p ≤ 0.0001, two way ANOVA with Sidak post-hoc test. The means and SD are shown in (**b**). **d** RT-qPCR quantification of the SARS-CoV-2 genomic N (gN), genomic E (gE) and subgenomic E (sgE) RNAs in lung homogenates of immunized hamsters on days 3 and 5 following challenge with SARS-CoV-2. The RNAs were quantified in triplicate by TaqMan assays and expressed as log_10_ copies per g of tissue (*n* = 5 per group per day, ***p ≤ 0.001 and ****p ≤ 0.0001, two way ANOVA with Sidak post-hoc test). Standard curves were generated using serial dilutions of plasmids containing the targeted sequence. The means and SD are shown. **e**, **f** Quantification, by limiting dilution on Vero E6 cells, of infectious SARS-CoV-2 in NTs (**e**) and lung homogenates (**f**) prepared from previously immunized hamsters harvested on the indicated days post-challenge (*n* = 5 per group, ****p < 0.0001, two way ANOVA with Sidak post-hoc test). The means and SD are shown.

In a continuation of Experiment #2 described above (see Fig. 4a for the timeline), we assessed protective efficacy against SARS-CoV-2 by challenging the hamsters on day 30 with 4.5 log_10_ TCID_50_ of SARS-CoV-2 WA1/2020 (Figs. 4a, 5).

Body weights of the APMV3- and APMV3/S-6P-immunized hamsters were monitored daily from day -1 to 5 post-challenge (Fig. 5a). Hamsters immunized with APMV3 lost weight from day 2 to 5 following SARS-CoV-2 challenge (7.3% average loss from their initial weights on day 0) while APMV3/S-6P-immunized hamsters continued to gain weight (3.3% average gain from their weight on day 0). No other clinical symptoms were observed in either group.

To compare inflammatory responses following SARS-CoV-2 challenge, the expression of 19 selected inflammatory/antiviral genes was analyzed by TaqMan real-time quantitative PCR (RT-qPCR) assays on total RNA extracted from lung homogenates (Fig. 5b, c). qPCR data were normalized to β-actin and expressed as the fold-increase over the mean values from three unimmunized and unchallenged hamsters. IFN-L and two IFN-regulated genes, CXCL10 and Mx2, were the three most strongly up-regulated genes on days 3 and 5 post-challenge in the lungs of APMV3-immunized animals (194-, 52- and 36-fold increase on average on day 3, respectively, Fig. 5b). In contrast, these three genes were barely up-regulated post-challenge in APMV3/S-6P-immunized hamsters (IFN-L did not have a detectable increase, and the average increases for CXCL10 and Mx2 were 2.2 and 1.6-fold, respectively). Analysis of the other 16 inflammatory/antiviral genes (Fig. 5c) similarly showed a strong induction of an inflammatory response post-challenge in the lungs of the APMV3-immunized hamsters, whereas the response post-challenge was much lower in the lungs of hamsters that had been immunized with APMV3/S-6P.

Next, we evaluated the presence of SARS-CoV-2 RNA in hamsters following challenge. SARS-CoV-2 genomic and subgenomic RNA were detected by RT-qPCR assays using RNA extracted from lung homogenates in the experiment described above (Fig. 5d). We used TaqMan assays to separately quantify genomic N (gN), genomic E (gE) RNA, and subgenomic E (sgE) mRNA. The presence of sgE mRNA reflects active SARS-CoV-2 transcription, indicative of effective SARS-CoV-2 replication. In APMV3/S-6P-immunized hamsters, the levels of SARS-CoV-2 gN and gE RNA were 4 log_10_ lower on days 3 and 5 post-challenge, compared to hamsters immunized with APMV3-empty-vector control. The level of SARS-CoV-2 sgE mRNA also was much lower in APMV3/S-6P-immunized hamsters as compared to APMV3-immunized hamsters (4 log_10_ difference on day 3), and close to the limit of detection (5.2 log_10_ copies/g). Thus, intranasal immunization of hamsters with APMV3/S-6P strongly restricted SARS-CoV-2 replication in the lungs.

Next, SARS-CoV-2 replication was evaluated by a limiting dilution assay on Vero E6 cells of NT and lung tissue homogenates collected on day 3 and 5 post-challenge (Fig. 5e–f). In the NTs of hamsters immunized with the APMV3-empty-vector, SARS-CoV-2 titers reached 5.2 log_10_ TCID_50_/g and 4.2 log_10_ TCID_50_/g on days 3 and 5 post-challenge, respectively (Fig. 5e), indicating that SARS-CoV-2 replicated efficiently. In contrast, no infectious SARS-CoV-2 was detected in the NTs of APMV3/S-6P-immunized hamsters on days 3 and 5 post-challenge. In the lungs of APMV3-empty-vector-immunized hamsters, SARS-CoV-2 titers reached 6.9 log_10_ TCID_50_/g and 5.1 log_10_ TCID_50_/g on day 3 and 5 post-challenge, respectively (Fig. 5f), indicating that the challenge virus replicated efficiently. However, SARS-CoV-2 was not detected by titration in the lungs of APMV3/S-6P-immunized hamsters at either day 3 or 5 post-challenge. In addition, no replication of SARS-CoV-2 was detected by titration from the brains of hamsters in any groups. Therefore, a single intranasal dose of APMV3/S-6P successfully prevented replication of SARS-CoV-2 in the upper and lower respiratory tract of hamsters and protected against severe lung inflammation and weight loss.

## Discussion

We developed a live-attenuated APMV3-based vaccine candidate against SARS-CoV-2 for intranasal delivery. The best-characterized avian paramyxovirus, APMV1 (also known as NDV), was previously shown to be well-tolerated in humans when used at high doses (usually administered parenterally) in cancer immunotherapy or as an experimental oncolytic agent^42^. Previous evaluation of NDV as a vaccine vector administered by the respiratory tract showed that it was highly attenuated in non-human primates due to its strong host-range restriction but nonetheless was highly immunogenic^43,44^. However, NDV is an important pathogen for poultry, and the use of the more-immunogenic NDV strains as vaccine vectors is not practical due to animal health concerns. APMV3 represents an alternative as a vector platform. Unlike NDV, APMV3 is only mildly pathogenic in poultry^30^. APMV3 is also highly attenuated in mammals due to strong host-range restriction^28^. Similar to other APMVs, APMV3 has a tropism for respiratory epithelial cells, and our results show that it readily infects and replicates in human lung epithelial A549 cells.

Preferably, a viral vector should be based on a virus for which humans have low seroprevalence. Otherwise, pre-existing immunity to the vector will likely restrict its infection and replication, thereby reducing its antigenic load and immunogenicity. For example, the use of adenoviruses as vectors depends on the availability of human or simian adenovirus serotypes that exhibit low immunity in the general human population^45,46^. APMV3 is very distinct from human viruses and it is anticipated to lack antigenic relatedness. In addition, human infection with APMV3 is generally unknown and likely resembles that of NDV, which occasionally infects individuals who have close contact with birds causing a restricted and mild infection^47^. Therefore, most humans have not been infected with APMV3 and are free of any pre-existing direct or cross-reacting immunity to APMV3. Following intranasal delivery, APMV3/S-6P is expected to replicate to low titers in superficial layers of the respiratory epithelium. Based on experience with human respiratory viruses, it is possible that anti-vector immunity induced by the first immunization with APMV3/S-6P will restrict vaccine “take” of a second intranasal dose, limiting the immunogenicity of the second dose. This would be a limitation if prime/boost approaches are needed. However, it is expected that heterologous prime/boost combinations of APMV3/S-6P, delivered intranasally, and an mRNA-based or adenovirus-vectored vaccine, delivered intramuscularly, will elicit very robust systemic and mucosal immune responses, effectively preventing SARS-CoV-2 infection and disease. Prime/boost combinations of APMV3/S-6P and mRNA-based SARS-CoV-2 vaccines will be evaluated in non-human primates.

In another approach, we recently developed a chimeric bovine/human parainfluenza type 3 (B/HPIV3) vector expressing the SARS-CoV-2 S protein as a pediatric vaccine candidate for intranasal immunization^38^. Like APMV3, B/HPIV3 is a replication-competent virus. However, an important difference is that B/HPIV3 is a vaccine candidate for the human pediatric pathogen HPIV3, and has been shown to be well-tolerated and immunogenic against HPIV3 in pediatric clinical trials^48,49^. Thus, B/HPIV3 expressing SARS-CoV-2 S provides a bivalent candidate vaccine against HPIV3 and COVID-19. However, the replication and immunogenicity of the B/HPIV3 vector would likely be restricted in HPIV3-immune individuals. Thus, the B/HPIV3 vector might not be effective in most adults, given the high seroprevalence to HPIV3, but should be suitable for pediatric use. In contrast, the APMV3 vector has potential use in both pediatric and adult populations regardless of exposure histories to human viruses.

In the present study, we expressed the full-length version (aa 1–1,273) of the S protein of the first available SARS-CoV-2 sequence from an ORF that was codon-optimized for human expression. The amino acid sequence of this S protein was identical to that of the challenge virus WA1/2020 that was used in this study. The S protein was engineered to be stabilized in the prefusion conformation in two versions (S-2P and S-6P) by the introduction of two (K986P and V987P) or six (K986P and V987P plus F817P, A892 P, A899P, and A942P) proline substitutions into the S2 domain, as previously described^12–15^. In addition, in each version, four amino acid residues of the furin cleavage site, 682-RRAR-685, were replaced with GSAS, as was previously described for secreted prefusion-stablized versions^11,12^. The S-2P protein is the form used in most of the SARS-CoV-2 vaccines in human use^13–15^ or in development^50^ and was shown to induce a higher level of neutralizing antibodies than the WT S^14^. The further inclusion of the four additional proline substitutions (to create S-6P) resulted in eight-fold higher production in eukaryotic cells in vitro and greater stability in the prefusion conformation, compared to S-2P^12^. Removing the furin cleavage site may further optimize the stabilization of the S protein in its prefusion conformation^51,52^, and was previously shown to increase the efficacy of a recombinant spike-based SARS-CoV-2 vaccine in the mouse model^53^. Furthermore, the S protein with the GSAS cleavage site instead of RRAR did not induce cell-cell fusion and syncytia formation^54^, as would be expected for a protein that is largely immobilized in the prefusion conformation. Thus, ablation of the furin cleavage site provides for an additional safeguard by rendering the SARS-CoV-2 S protein non-functional for mediating fusion in a live viral vector. In our study, we did not detect any replication of APMV3 or APMV3/S-6P outside of the respiratory tract in hamsters, indicating that the tissue tropism of the APMV3 vector expressing S did not differ from that of APMV3 in this model.

APMV3 vectors were constructed to express the WT S, S-2P, or S-6P proteins, and all viruses were efficiently recovered and expressed the corresponding S protein when passaged in Vero cells. However, when evaluated by plaque assay following replication to high titer in embryonated chicken eggs, we found that a high proportion of APMV3/S and APMV3/S-2P virions had lost the expression of WT S and S-2P proteins, respectively. In contrast, expression of S-6P by APMV3/S-6P was maintained in a high percentage of virions, even though this virus replicated similarly to high titers in eggs. The genetic instability of the APMV3/S and APMV3/S-2P stocks precluded direct comparison with APMV3/S-6P. The reasons for the greater genetic stability of APMV3/S-6P remain to be elucidated. As one possibility, we found that the S-6P protein was incorporated in the APMV3/S-6P virion (and the S-2P form of S likely was also incorporated into the virions), and it may be that incorporation of the less-stable S-2P protein was more detrimental to APMV3 virion formation than the more-stable S-6P protein, and thus conferred a greater selective pressure for loss of expression of its gene.

We further pursued the characterization of APMV3/S-6P in vitro. The replication kinetics of APMV3/S-6P were comparable to those of WT APMV3 in Vero and A549 cells, but the overall replication in A549 cells was lower. However, the ability to grow APMV3/S-6P to high titers in embryonated chicken eggs, an approved vaccine substrate, would facilitate vaccine manufacture. Assuming that 6 log_10_ PFU would be selected as a vaccine dose and that five to 10 ml of virus can be harvested from one egg, about 500 to 1,000 doses per egg could be generated in two days.

As noted, the S-6P protein was incorporated into the APMV3 virion. This likely did not confer any advantage to the vector, since the S-6P protein should be largely non-functional. However, in a previous study, we found that efficient incorporation of the respiratory syncytial virus (RSV) fusion (F) protein into the B/HPIV3 virion resulted in an increase in the quantity and quality of RSV-neutralizing antibodies that was comparable to that provided by stabilization in the prefusion conformation without incorporation^55^. The increased immunogenicity associated with incorporation into the vector particle might be due to a more efficient presentation to the immune system of a protein that is in a multimeric array and/or is in a particle^55^. In any event, the incorporation of S-6P into the APMV3 vector particles that occurred might similarly increase its immunogenicity.

In the hamster model, APMV3/S-6P replicated as efficiently as APMV3 in the upper respiratory tract, but was slightly reduced in the lungs on day 3 pi, suggesting that the supernumerary gene marginally reduced the replication efficiency in this model. Despite this mild restriction in the lung, a single intranasal delivery of APMV3/S-6P was highly immunogenic in hamsters. Indeed, 6 log_10_ PFU of APMV3/S-6P induced robust serum neutralizing antibody titers against the lineage A isolate WA1/2020 in all immunized hamsters. We detected a strong serum IgG response to the secreted version of prefusion S and to its RBD domain (with mean titers above 5.4 log_10_ in two independent studies). This compares well with, and is considered to be at least as potent as, responses observed in animals that had been vaccinated intranasally or intramuscularly with adenovirus-vectored SARS-CoV-2 vaccines^56–58^. The APMV3/S-6P-induced antibodies also neutralized two variants of concern of lineages B.1.1.7 (Alpha) and B.1.351 (Beta), although the neutralizing titers to a representative of the Beta variant were reduced and more variable among hamsters, similar to findings with other vaccine candidates expressing S proteins of representatives of the SARS-CoV-2 lineage A^59^. Thus, APMV3/S-6P would be expected to generate at least some level of protection against these variants of concern.

APMV3/S-6P efficiently protected hamsters against challenge with SARS-CoV-2. Unlike hamsters previously immunized with the empty APMV3 vector, APMV3/S-6P-immunized hamsters were protected from weight loss after SARS-CoV-2 challenge. The low or absent inflammatory response in the lungs of APMV3/S-6P-immunized hamsters following challenge further confirmed efficient protection against challenge. Furthermore, the absent or very low levels of infectious SARS-CoV-2 in the NTs and lungs suggested that intranasal inoculation with a single dose of APMV3/S-6P induced near-sterilizing immunity in the hamster model. Correlation of SARS-CoV-2-neutralizing serum antibodies with protective efficacy was described in an earlier report^14^. However, since the present vaccine candidate was live and was administered by the intranasal route, it is reasonable to suggest that a local airway IgA response as well as systemic and local T cell-mediated responses also contributed to protection in the respiratory tract. Since it is technically challenging to reliably determine antibody titers in the mucosal lining fluid in hamsters, we measured IgA titers to the SARS-CoV-2 S protein in serum by an enhanced IgA ELISA, showing that a single intranasal dose of APMV3/S-6P induced a potent IgA response. A more extensive comparison of the mucosal and the serum IgA response to immunization with APMV3 vectors via the respiratory route will be performed in future rhesus macaques studies.

Intranasal vaccination stimulates local mucosal immunity in addition to systemic immunity^60^. In contrast, all currently approved human COVID-19 vaccines are administered parenterally and thus do not directly stimulate respiratory tract immunity. It is generally recognized that local mucosal immunity is particularly effective in restricting virus replication and re-infection in the respiratory tract. For example, studies with experimental vaccines for RSV in animal models showed that live-attenuated vaccines administered by the intranasal route provided greater protection in the upper respiratory tract compared to parenterally administered vaccinia virus vectors or RSV subunits^61,62^. Systemic immune effectors may not efficiently access the respiratory tract, especially the upper respiratory tract. For example, while serum IgG accesses the respiratory tract by transudation, for IgG specific to influenza A virus there was a gradient of approximately 350:1 between titers in the serum versus the upper respiratory tract^63^. Immunization in the respiratory tract induces local B cell responses in nasal- and bronchus-associated lymphoid tissue (NALT and BALT), resulting in the induction of mucosally secreted and serum IgA and IgG, as observed in recent studies with adenovirus-vectored SARS-CoV-2 vaccine candidates^56,58^. Respiratory immunization also induces local and systemic T cell responses whereas parenteral immunization primarily induces systemic responses. For example, respiratory immunization with a parainfluenza virus vector expressing the Ebola virus GP efficiently induced lung-resident CD8+ and CD4+ T cells, compared to a predominantly systemic response to Ebola virus GP expressed by an alphavirus replicon given intramuscularly^64^. Similarly, an intranasal vectored SARS-CoV-2 vaccine induced tissue-resident T cell responses, thereby protecting the upper and lower respiratory tract against SARS-CoV-2^65^. In a recent study^66^, immunization of hamsters by the parenteral route with a prime-boost regimen of Moderna mRNA-1273 SARS-CoV-2 vaccine, followed by challenge with SARS-CoV-2, resulted in a high level of restriction of challenge SARS-CoV-2 replication in the lungs, whereas there was substantial replication of challenge SARS-CoV-2 in the upper respiratory tract. Thus, while replication in the lung was efficiently restricted by parenteral vaccination, replication in the upper respiratory tract was not efficiently restricted, which would increase shedding and virus transmission^66^. Therefore, SARS-CoV-2 vaccines capable of direct immunization of the respiratory tract are of interest.

In summary, we generated an attenuated, replication-competent APMV3-vectored vaccine that stably expresses a version of the SARS-CoV-2 S protein that has been stabilized in the prefusion conformation by six proline substitutions. The vaccine virus replicates to high titers in embryonated chicken eggs, which are a suitable substrate for vaccine manufacture. This vector provides for direct immunization of the respiratory tract using a virus for which humans generally lack immunity that otherwise could interfere with vaccination. A single intranasal dose of this vaccine candidate in hamsters elicited robust serum IgG and IgA antibody responses against SARS-CoV-2 S and conferred full protection of both the upper and lower respiratory tract against SARS-CoV-2 challenge. These results support the further clinical development of this intranasal vaccine candidate. This could be used as a stand-alone vaccine or a heterologous prime-boost with any of the vaccines presently in human use and in development.

## Methods

### Cells

Human lung epithelial A549 (ATCC CCL-185) cells were cultured in F-12K (ATCC) with 10% fetal bovine serum (FBS, GE Healthcare). Baby hamster kidney cells expressing T7 RNA polymerase (BSR T7/5) were grown in Glasgow minimum essential medium (MEM) (Thermo Fisher Scientific) with 10% FBS, 1% l-glutamine (Thermo Fisher Scientific), and 2% MEM Amino Acids (Thermo Fisher Scientific). African green monkey kidney Vero (ATCC CCL-81) and Vero E6 (ATCC CRL-1586) cells were cultured in DMEM + GlutaMAX (Thermo Fisher Scientific) supplemented with 5% FBS and 1% L-glutamine. Vero E6 cells are suitable for propagating and titrating SARS-related coronaviruses due to high ACE2 expression^67,68^. Vero E6 cells stably expressing human TMPRSS2 that further improve SARS-CoV-2 replication^69^ were generated using the Sleeping Beauty transposase system^38^. All experiments and assays in cell culture were performed at 37 °C.

### Generation of APMV3 expressing SARS-CoV-2 S

A cDNA clone encoding the antigenome of APMV3 (Parakeet/Netherlands/449/75^29^) and N, P, and L support plasmids were used in this study^32,33^. The ORF encoding the full-length WT SARS-CoV-2 S derived from the first available sequence (GenBank MN908947) was codon-optimized for human usage and three versions were designed: WT S, S-2P, and S-6P (see Results and Fig. 1). The WT S, S-2P, and S-6P cDNAs were designed to be flanked on the 3’-ends by a *Sac*II restriction enzyme site followed by a Kozak sequence, and on the 5’-ends by a copy of the P gene-end sequence, followed by a copy of the M-F intergenic sequence, an APMV3 gene start (which is identical for each APMV3 ORF) and a second *Sac*II site. The cDNA lengths were designed to maintain the “rule of six”^70^. The codon-optimized WT version of S was synthesized commercially (BioBasic), and stabilizing mutations were introduced by site-directed mutagenesis (QuikChange Multi Site-Directed Mutagenesis Kit, Agilent). Each version of S was inserted into the unique *Sac*II site in the 3’ noncoding region of the APMV3 M gene (at nt 3,222; Fig. 1a). The encoded viruses APMV3/S, APMV3/S-2P, and APMV3/S-6P were rescued by reverse genetics on BSR T7/5 cells^36^ and passaged once or twice on Vero cells to generate passage 2 (P2) or passage 3 (P3) virus stocks. The parental empty APMV3 vector was also rescued and passaged once on Vero cells.

Two independent stocks of APMV3, APMV3/S and APMV3/S-2P and three independent stocks of APMV3/S-6P were generated by infecting 11-day-old specific pathogen-free, embryonated chicken eggs (Charles River Laboratories). Specifically, 200–2,000 PFUs of the P2 or P3 virus stocks were inoculated into the allantoic cavities of 5 to 30 eggs per stock. Allantoic fluids were harvested on day 2 post-infection and samples from individuals eggs were screened for hemagglutination by standard hemagglutination (HA) assay using a 0.5% suspension of chicken red blood cells (Lampire). For each independent stock, allantoic fluids with HA titers equal to or higher than 1:512 for WT APMV3 and equal to or higher than 1:64 for the S-expressing APMV3s were pooled and clarified by centrifugation at 850 × *g* for 10 min at 4 °C. Supernatants were aliquoted, snap-frozen on dry ice, and stored at −80 °C until further use.

### APMV3 titration by immunoplaque assay and dual-staining immunoplaque assay

Sub-confluent Vero cells in 24-well plates were infected in duplicate with ten-fold serially diluted viruses. After 2 h incubation at 37 °C, cells were washed with Opti-MEM (Thermo Fisher Scientific) and overlaid with 1 ml per well of 0.8% methylcellulose dissolved in Opti-MEM containing 1% L-glutamine, 2.5% penicillin-streptomycin, 0.1% gentamicin, 0.5% amphotericin B and 2% TrypLE Select (Thermo Fisher Scientific). On day 4 post-infection, cells were fixed using ice-cold 80% methanol. For dual-staining assays to detect expression of APMV3 and SARS-CoV-2 antigens, wells were incubated for 2 h at room temperature (RT) with a primary chicken anti-APMV3 serum (1:2,000) and a human anti-SARS-CoV-2 S RBD antibody (CR3022, 1:2,000) (or, as a negative control, was replaced with a combination of normal chicken IgY control (R&D Systems, cat# AB-101-C, 1:1,000) and human IgG1 isotype control (BioLegend, cat# 403501, 1:1,000)). After extensive washing with PBS, wells were incubated for 1 h at RT with 1:2,000 diluted infrared dye (IRDye)-conjugated 680LT donkey anti-chicken IgY (LI-COR, cat# 926-68028) and IRDye 800CW goat anti-human IgG secondary antibodies (LI-COR, cat# 926-32232). After washing with PBS, plates were scanned with an Odyssey CLx imager (LI-COR).

### Amplification and sequencing of SARS-CoV-2 virus stocks

The SARS-CoV-2 USA-WA1/2020 isolate (lineage A; GenBank MN985325; GISAID: EPI_ISL_404895; obtained from Dr. Natalie Thornburg *et al*., Centers for Disease Control and Prevention (CDC)) was passaged twice on Vero E6 cells. The amino acid sequence of the S protein of WA1/2020 (GenBank MN985325) is identical to that of the first available SARS-CoV-2 sequence that we used to design APMV3/S (GenBank MN908947). Because WA1/2020 contains an S sequence homologous to the sequence from which S-2P and S-6P were derived, we used WA1/2020 as challenge virus in this study. The USA/CA_CDC_5574/2020 isolate [lineage B.1.1.7 (Alpha); GISAID: EPI_ISL_751801; obtained from CDC] and the USA/MD-HP01542/2021 isolate [lineage B.1.351 (Beta); GISAID: EPI_ISL_890360; obtained from Dr. Andrew Pekosz, Johns Hopkins University] were passaged on TMPRSS2-expressing Vero E6 cells. The SARS-CoV-2 stocks were titrated in Vero E6 cells by determination of the 50% tissue culture infectious dose (TCID_50_)^71^. These viruses were included to evaluate the breadth of the immune response induced by the APMV3 vectors. The complete-genome sequences of the SARS-CoV-2 WA1/2020 challenge virus and the representative of lineage B.1.1.7 (Alpha) and B.1.351 (Beta) were determined by Illumina deep-sequencing, confirming that the S ORFs of the SARS-CoV-2 challenge virus pool and the representatives of the Alpha and Beta variants were identical to that of their consensus sequences. All experiments with SARS-CoV-2 were performed in biosafety level 3 (BSL3) containment laboratories approved by the USDA and CDC.

### Antibodies and antigens

We produced a rabbit antiserum against sucrose-purified APMV3 using a subcutaneous chamber method^72^. We also produced a goat anti-S hyperimmune serum N25-154 using a secreted form of S-2P protein that was expressed in Expi293 cells and purified by affinity chromatography and size-exclusion chromatography^38^. cDNA plasmids encoding the light and heavy chains of the anti-S-RBD human monoclonal IgG antibody CR3022 were generously provided by Drs. Peter Kwong and Baoshan Zhang (Vaccine Research Center, National Institute of Allergy and Infectious Diseases (NIAID), National Institutes of Health (NIH)). Expi293 suspension cells grown at 37 °C with 10% CO_2_ in a shaking incubator were co-transfected with these plasmids following the manufacturer’s recommendations. The CR3022 monoclonal antibody was purified by affinity chromatography at 5 to 6 days post-transfection. Aliquots were snap-frozen in liquid nitrogen and stored at −80 °C.

The secreted prefusion-stabilized form of SARS-CoV-2 S (2019-nCoV S-2P_dFurin_F3CH2S, aa 1–1,208^11^) was expressed in Expi293 cells from a plasmid generously provided by Drs. Barney Graham, Kizzmekia Corbett (Vaccine Research Center, NIAID, NIH), and Jason McLellan (University of Texas at Austin), and purified by affinity chromatography and size-exclusion chromatography^38^. A fragment of the S protein containing the RBD region (aa 328–531) was expressed in Expi293 cells from a cDNA plasmid obtained from Dr. David Veesler (University of Washington) through BEI Resources, NIAID, NIH^73^. Aliquots were snap-frozen in liquid nitrogen and stored at −80 °C.

### Western blotting

Vero or A549 cells seeded in 6-well plates were infected with APMV3 or APMV3/S-6P at an MOI of 0.01 pfu/cell. At the indicated time points, cells from replica wells were washed once with PBS and lysed with Bolt LDS Sample Buffer (Thermo Fisher Scientific) containing Halt Protease Inhibitor Cocktail (Thermo Fisher Scientific). Then, lysates were passed through QIAshredder columns (Qiagen) and stored at −80 °C. Parts of the clarified cell lysates were denatured with Bolt LDS Sample Buffer and Sample Reducing Agent (Thermo Fisher Scientific) at 90 °C for 5 min. Denatured proteins were resolved on 4–12% Bis-Tris gels (Thermo Fisher Scientific) and transferred to polyvinylidene difluoride membranes. Membranes were blocked with Intercept Blocking Buffer and probed with the following primary and secondary antibodies diluted in Intercept T20 Antibody Diluent (LI-COR): rabbit anti-APMV3 serum (1:2,000); goat anti-SARS-CoV-2 S serum (1:5,000); mouse anti-β-actin antibody (R&D Systems, cat# MAB8929,1:10,000) and IRDye 680RD donkey anti-rabbit IgG (LI-COR, cat# 926-68073, 1:10,000); IRDye 800CW donkey anti-goat IgG (LI-COR, cat# 926-32214, 1:10,000); IRDye 800CW donkey anti-mouse IgG (LI-COR, cat# 926-32212, 1:10,000). The membranes were washed and scanned using an Odyssey CLx imager. Fluorescence signals of the SARS-CoV-2 S and APMV3 N proteins were background-corrected automatically by the Image Studio Lite software (LI-COR) and measured to quantify the intensity of each protein band. Values were normalized in each gel to β-actin intensity of the same sample. The uncropped and unprocessed image that was used to generate Fig. 2a is shown in supplementary Fig. S5.

### Flow cytometry

Mock- or virus-infected Vero or A549 cells in 6-well plates were trypsinized with TrypLE Select and harvested at the indicated time points. Next, cells were stained with LIVE/DEAD Fixable Near-IR Dead Cell Stain Kit (Thermo Fisher Scientific) and fixed with Cytofix/Cytoperm (BD Biosciences). After permeabilization in Perm/Wash buffer (BD Biosciences), cells were incubated with a phycoerythrin (PE)-conjugated human anti-SARS-CoV-2 S monoclonal antibody (CR3022) (1:100) in Perm/Wash buffer for 20 min in the dark. Stained cells were washed five times with Perm/Wash buffer and resuspended in PBS until analyzed by a FACSymphony Flow Cytometer (BD Biosciences). At least 20,000 events were acquired for each sample. Data were analyzed with FlowJo (version 10.8). First, the quality control of each acquired sample was performed using the FlowAI plugin^74^. Then, compensation was performed automatically using beads for each antibody. Live/dead staining, forward scatter height, and forward scatter area were used to identify single live cells. The expression of the SARS-CoV-2 S protein was analyzed on single live cells.

### Silver staining of sucrose-purified APMV3s

Preparations of egg-grown APMV3 and APMV3/S-6P viruses were subjected to centrifugation on 30–60% sucrose step gradients at 134,000 × *g* for 2 h at 4 °C. The opaque virus bands were collected, diluted in Tris-EDTA-NaCl (TEN) buffer, and pelleted by centrifugation at 8000 × *g* for 2 h at 4 °C. The pelleted virus was resuspended with TEN buffer, aliquoted, and stored at −80 °C. Protein concentrations were determined by bicinchoninic acid (BCA) assay using a Micro BCA Protein Assay Kit (Thermo Fisher Scientific). Three micrograms of each preparation were denatured with Bolt LDS Sample Buffer and Sample Reducing Agent and boiled at 90 °C for 5 min. Proteins were separated on 4–12% Bis–Tris gels and stained using a Pierce Silver Stain Kit (Thermo Fisher Scientific) following the manufacturer’s instructions.

### Electron microscopy of sucrose-purified virus preparations

Two ml of egg-grown APMV3 or APMV3/S-6P were incubated with goat anti-SARS-CoV-2 S serum at 1:50 for 1 h under agitation. Mixtures were loaded on a 30–60% sucrose gradient in 5 mL thin-wall ultra-clear tubes (Beckman) and centrifugated at 160,000 × *g* at 4 °C for 2 h. Virus bands were collected and fixed with 2% paraformaldehyde in PBS. For immunogold staining, 10 μl of samples were adsorbed, in saturated humid chambers at RT, to freshly glow-discharged 200 mesh Formvar/carbon-coated Ni grids for 30 min. Grids were washed in PBS, then blocked with 2% bovine serum albumin (BSA) in PBS followed by 0.1% acetylated BSA (BSA-c, Aurion) in PBS. All samples were labeled with donkey anti-goat antibody conjugated to 6 nm Au (Aurion, cat# 25803) that was 1:50 diluted in 0.1% BSA-c in PBS as per the manufacturer’s instructions. The grids were washed with 0.1% BSA-c in PBS, then with PBS, and then in water followed by negative-staining with methylamine vanadate and electron microscopy observation.

### Hamster studies

The hamster studies were approved by the NIAID Animal Care and Use Committee. All the animal experiments were carried out following the Guide for the Care and Use of Laboratory Animals by the NIH. Six-week-old golden Syrian hamsters (*Mesocricetus auratus*, Envigo Laboratories, Frederick, MD) were used in two separate experiments conducted in BSL2 and BSL3 facilities approved by the USDA and CDC. Intranasal immunization or challenges were performed under light isoflurane anesthesia. Animals were euthanized by CO_2_ inhalation prior to necropsy.

In Experiment #1, six-week-old female Syrian hamsters, randomly divided into two groups of 45 animals each, were bled for serology and were immunized intranasally 11 days later with 6 log_10_ PFU of either APMV3 or APMV3/S-6P diluted in Leibovitz’s L-15 medium (50 µl per nostril). On days 3, 5, and 7 pi, six animals per group were necropsied and brain, blood, nasal turbinate, lung, liver, kidney, spleen, and intestine were harvested. Tissues were weighed, mixed with L-15 medium (10 ml per g of tissue), homogenized using a gentleMACS Dissociator (Miltenyi Biotech), and clarified by centrifugation. Aliquots were snap-frozen to be stored at −80 °C. Virus replication was evaluated by dual-staining immunoplaque assay, as described above. Two animals per group were necropsied on days 3, 5, and 7 for IHC. Blood was collected for serology on day 26 pi from the remaining animals (*n* = 21 per group).

In Experiment #2, six-week-old male Syrian hamsters were randomly divided into two groups of 10 animals each. Animals were bled for serology on day −1. On day 0, the animals were immunized intranasally with 6 log_10_ PFU of either APMV3 or APMV3/S-6P. On day 27 pi, sera were collected and animals were transferred to the BSL3 facility. On day 30 pi, animals were challenged intranasally with 4.5 log_10_ TCID_50_ of homologous SARS-CoV-2 WA1/2020 virus in 100 µl volumes per animal. Body weights and clinical symptoms were monitored daily. On days 3 and 5 post-challenge, five animals per group were necropsied, and nasal turbinates, lungs, and brains were collected, and processed as described above. The presence of the challenge virus in clarified tissue homogenates was evaluated by limiting dilution titration on Vero E6 cells. Titers were expressed as 50% tissue culture infectious dose (TCID_50_) per g tissue^71^.

### Immunohistopathology analysis

Lung tissue samples from hamsters were fixed in 10% neutral buffered formalin, processed through a Leica ASP6025 tissue processor (Leica Biosystems), and embedded in paraffin. Five-micrometer tissue sections were stained with hematoxylin and eosin for routine histopathology. For IHC evaluation, sections were deparaffinized and rehydrated. After epitope retrieval, sections were incubated with goat hyperimmune serum to SARS-CoV-2 S (N25-154) at 1:1,000, and rabbit polyclonal anti-APMV3 serum at 1:100. Chromogenic staining was carried out on the Bond RX platform (Leica Biosystems) according to manufacturer-supplied protocols. Detection with DAB chromogen was completed using the Bond Polymer Refine Detection kit (Leica Biosystems). The VisUCyte anti-goat horse radish peroxidase (HRP) polymer (R&D Systems) replaced the standard Leica anti-rabbit HRP polymer from the kit to bind the goat anti-SARS-CoV-2 S antibodies. Slides were finally cleared through gradient alcohol and xylene washes prior to mounting. Sections were examined by a board-certified veterinary pathologist using an Olympus BX51 light microscope and photomicrographs were taken using an Olympus DP73 camera.

### SARS-CoV-2 neutralization assay

SARS-CoV-2-neutralizing antibody titers were determined in the BSL3 laboratory. Heat-inactivated sera were 2-fold serially diluted in Opti-MEM and mixed with an equal volume of SARS-CoV-2 (100 TCID_50_) and incubated at 37 °C for 1 h. Mixtures were added to quadruplicate wells of Vero E6 cells in 96-well plates and incubated for four days. The 50% neutralizing dose (ND_50_) was defined as the highest dilution of serum that completely prevented cytopathic effect in 50% of the wells and was expressed as a log_10_ reciprocal value.

### APMV3 neutralization assay

Heat-inactivated hamster sera were four-fold serially diluted and mixed with an equal volume of APMV3 (1,000 PFU) and incubated at 37 °C for 30 min. Then, the mixture was added to duplicate wells of Vero cells that were seeded on 24-well plates. After 1 h incubation at 37 °C, a methylcellulose overlay including supplements as described was added to each well. Four days later, plates were fixed and immunostained for APMV3 as described above. The 60% plaque reduction neutralization titer (PRNT_60_) was defined as the highest serum dilution that showed a 60% reduction in the number of plaques compared to the number counted in wells without serum and was expressed as a log_10_ reciprocal value.

### Enzyme-linked immunosorbent assay (ELISA)

Nunc Maxisorp flat-bottom, 96-well plates (Thermo Fisher Scientific) were coated with purified antigens (SARS-CoV-2 S or SARS-CoV-2 S RBD) diluted in carbonate bicarbonate buffer (Sigma) at 4 °C overnight, and ELISAs were performed^38^. Plates were read at 450 nm using a microplate reader (Synergy Neo2, BioTek). For each serum, the average optical density (OD) at each dilution from duplicate wells was calculated with the average OD in blank wells subtracted. An end-point titer was defined as the highest dilution of each serum corresponding to the OD above the cutoff (average OD in blank wells plus three standard deviations) and was determined by interpolation using a model standard curve (Sigmoidal, 4PL, X is log [concentration]) in Prism 8 (GraphPad Software) and expressed as a log_10_ reciprocal value. Serum IgA antibodies to the secreted form of S-2P or RBD were measured by DELFIA-TRF (Perkin Elmer) following the supplier’s protocol.

### Analysis of gene expression and quantification of SARS-CoV-2 RNA in lung tissues of hamsters

From 0.125 ml of lung homogenates (0.1 g of tissue per ml), total RNA was isolated using TRIzol Reagent, Phasemaker Tubes Complete System (Thermo Fisher Scientific), and PureLink RNA Mini Kit (Thermo Fisher Scientific) following the manufacturer’s instructions. Using the High-Capacity RNA-to-cDNA Kit (Thermo Fisher Scientific), cDNA was amplified from 350 ng of each RNA. These cDNAs were used to quantify the level of expression of inflammatory/antiviral genes as well as SARS-CoV-2 RNA.

TaqMan low-density array cards (Thermo Fisher Scientific) were designed to contain TaqMan primers and probes for golden Syrian hamster’s inflammatory/antiviral genes and β-actin as a housekeeping gene^8,75–77^. Each cDNA was mixed with TaqMan Fast Advanced Master Mix (Thermo Fisher Scientific) and added into each fill port of the array cards for RT-qPCR with QuantStudio 7 Pro (Thermo Fisher Scientific). Results were analyzed using the comparative threshold cycle (ΔΔC_T_) method, normalized to β-actin, and expressed as fold changes over the average expression in three non-immunized, non-challenged hamsters. Heat maps were generated using the Gene Expression Similarity Investigation Suite (GENESIS program, release 1.8.1, http://genome.tugraz.at).

Quantification of genomic N (gN), E (gE), and subgenomic E mRNA (sgE) of the SARS-CoV-2 challenge virus WA1/2020 was done in triplicate using TaqMan Fast Advanced Master Mix. The PCR strategy was taken from published work^78–80^. The sgE assay used a forward PCR primer in the leader sequence and a reverse primer in the E gene, which thus made amplification specific to that subgenomic RNA, while the gE and gN assay used forward and reverse primers in the coding sequence, which thus made amplification specific to genomic RNA. Standard curves were generated using pcDNA3.1 plasmids containing gN, gE, or sgE sequences. The sensitivity of these TaqMan assays was 10 copies, which corresponds to a limit of detection of 5.2 log_10_ copies per g of lung tissue.

### Statistical analysis

Datasets were assessed for significance using Mann-Whitney tests, Mixed effects analysis, one-way or two-way ANOVA with Tukey’s or Sidak’s post-hoc tests using Prism 9 (GraphPad Software). Data were only considered significant at *p* < 0.05.

### Reporting summary

Further information on research design is available in the Nature Research Reporting Summary linked to this article.

## Supplementary information

Supplementary material

Reporting Summary

## Data Availability

The datasets generated and/or analyzed during the current study are available within the main and supplemental figures. All data is available from the corresponding authors upon request.

## References

[1] El Sahly HM (2021). Efficacy of the mRNA-1273 SARS-CoV-2 vaccine at completion of blinded phase. N. Engl. J. Med..

[2] Thomas SJ (2021). Safety and efficacy of the BNT162b2 mRNA Covid-19 vaccine through 6 months. N. Engl. J. Med..

[3] Sadoff J (2022). Final analysis of efficacy and safety of single-dose Ad26.COV2.S. N. Engl. J. Med..

[4] Hoffmann M (2020). SARS-CoV-2 cell entry depends on ACE2 and TMPRSS2 and is blocked by a clinically proven protease inhibitor. Cell.

[5] Zhou P (2020). A pneumonia outbreak associated with a new coronavirus of probable bat origin. Nature.

[6] Ju B (2020). Human neutralizing antibodies elicited by SARS-CoV-2 infection. Nature.

[7] Braun J (2020). SARS-CoV-2-reactive T cells in healthy donors and patients with COVID-19. Nature.

[8] Sanchez-Felipe L (2021). A single-dose live-attenuated YF17D-vectored SARS-CoV-2 vaccine candidate. Nature.

[9] Lu, M. et al. A safe and highly efficacious measles virus-based vaccine expressing SARS-CoV-2 stabilized prefusion spike. *Proc. Natl Acad. Sci. USA* **118**, 10.1073/pnas.2026153118 (2021). 10.1073/pnas.2026153118 PMC8000430 33688034

[10] Pallesen J (2017). Immunogenicity and structures of a rationally designed prefusion MERS-CoV spike antigen. Proc. Natl Acad. Sci. USA.

[11] Wrapp D (2020). Cryo-EM structure of the 2019-nCoV spike in the prefusion conformation. Science.

[12] Hsieh CL (2020). Structure-based design of prefusion-stabilized SARS-CoV-2 spikes. Science.

[13] Corbett KS (2020). SARS-CoV-2 mRNA vaccine design enabled by prototype pathogen preparedness. Nature.

[14] Mercado NB (2020). Single-shot Ad26 vaccine protects against SARS-CoV-2 in rhesus macaques. Nature.

[15] Walsh EE (2020). Safety and immunogenicity of two RNA-based Covid-19 vaccine candidates. N. Engl. J. Med..

[16] Brown CM (2021). Outbreak of SARS-CoV-2 infections, including COVID-19 vaccine breakthrough infections, associated with large public gatherings—Barnstable County, Massachusetts, July 2021. MMWR Morb. Mortal. Wkly. Rep..

[17] Bergwerk M (2021). Covid-19 breakthrough infections in vaccinated health care workers. N. Engl. J. Med..

[18] Rima B (2019). ICTV virus taxonomy profile: paramyxoviridae. J. Gen. Virol..

[19] DiNapoli JM (2007). Newcastle disease virus, a host range-restricted virus, as a vaccine vector for intranasal immunization against emerging pathogens. Proc. Natl Acad. Sci. USA.

[20] Hu, Z., Ni, J., Cao, Y. & Liu, X. Newcastle disease virus as a vaccine vector for 20 years: a focus on maternally derived antibody interference. *Vaccines (Basel)* **8**, 10.3390/vaccines8020222 (2020). 10.3390/vaccines8020222 PMC7349365 32422944

[21] Bukreyev A (2005). Recombinant newcastle disease virus expressing a foreign viral antigen is attenuated and highly immunogenic in primates. J. Virol..

[22] DiNapoli JM (2010). Respiratory tract immunization of non-human primates with a Newcastle disease virus-vectored vaccine candidate against Ebola virus elicits a neutralizing antibody response. Vaccine.

[23] Freeman AI (2006). Phase I/II trial of intravenous NDV-HUJ oncolytic virus in recurrent glioblastoma multiforme. Mol. Ther..

[24] Pecora AL (2002). Phase I trial of intravenous administration of PV701, an oncolytic virus, in patients with advanced solid cancers. J. Clin. Oncol..

[25] DiNapoli JM (2010). Newcastle disease virus-vectored vaccines expressing the hemagglutinin or neuraminidase protein of H5N1 highly pathogenic avian influenza virus protect against virus challenge in monkeys. J. Virol..

[26] Martinez-Sobrido L (2006). Protection against respiratory syncytial virus by a recombinant Newcastle disease virus vector. J. Virol..

[27] Sun, W. et al. A Newcastle Disease Virus (NDV) expressing a membrane-anchored spike as a cost-effective inactivated SARS-CoV-2 vaccine. *Vaccines (Basel)* **8**, 10.3390/vaccines8040771 (2020). 10.3390/vaccines8040771 PMC7766959 33348607

[28] Samuel AS, Subbiah M, Shive H, Collins PL, Samal SK (2011). Experimental infection of hamsters with avian paramyxovirus serotypes 1 to 9. Vet. Res..

[29] Alexander DJ (2000). Newcastle disease and other avian paramyxoviruses. Rev. Sci. Tech..

[30] Kumar S, Militino Dias F, Nayak B, Collins PL, Samal SK (2010). Experimental avian paramyxovirus serotype-3 infection in chickens and turkeys. Vet. Res..

[31] Yoshida A (2019). Novel avian paramyxovirus-based vaccine vectors expressing the Ebola virus glycoprotein elicit mucosal and humoral immune responses in guinea pigs. Sci. Rep..

[32] Yoshida A, Samal SK (2017). Avian paramyxovirus type-3 as a vaccine vector: identification of a genome location for high level expression of a foreign gene. Front. Microbiol..

[33] Shirvani E, Varghese BP, Paldurai A, Samal SK (2020). A recombinant avian paramyxovirus serotype 3 expressing the hemagglutinin protein protects chickens against H5N1 highly pathogenic avian influenza virus challenge. Sci. Rep..

[34] Wu F (2020). A new coronavirus associated with human respiratory disease in China. Nature.

[35] Rambaut A (2020). A dynamic nomenclature proposal for SARS-CoV-2 lineages to assist genomic epidemiology. Nat. Microbiol..

[36] Buchholz UJ, Finke S, Conzelmann KK (1999). Generation of bovine respiratory syncytial virus (BRSV) from cDNA: BRSV NS2 is not essential for virus replication in tissue culture, and the human RSV leader region acts as a functional BRSV genome promoter. J. Virol..

[37] Kumar S, Nayak B, Collins PL, Samal SK (2008). Complete genome sequence of avian paramyxovirus type 3 reveals an unusually long trailer region. Virus Res.

[38] Liu, X. et al. A single intranasal dose of a live-attenuated parainfluenza virus-vectored SARS-CoV-2 vaccine is protective in hamsters. *Proc Natl Acad Sci USA* **118**, 10.1073/pnas.2109744118 (2021). 10.1073/pnas.2109744118 PMC8685679 34876520

[39] Chan JF (2020). Simulation of the clinical and pathological manifestations of Coronavirus Disease 2019 (COVID-19) in golden Syrian hamster model: implications for disease pathogenesis and transmissibility. Clin. Infect. Dis..

[40] Imai M (2020). Syrian hamsters as a small animal model for SARS-CoV-2 infection and countermeasure development. Proc. Natl Acad. Sci. USA.

[41] Sia SF (2020). Pathogenesis and transmission of SARS-CoV-2 in golden hamsters. Nature.

[42] Burman, B., Pesci, G. & Zamarin, D. Newcastle Disease Virus at the forefront of cancer immunotherapy. *Cancers (Basel)* **12**, 10.3390/cancers12123552 (2020). 10.3390/cancers12123552 PMC7761210 33260685

[43] DiNapoli JM (2009). Delivery to the lower respiratory tract is required for effective immunization with Newcastle disease virus-vectored vaccines intended for humans. Vaccine.

[44] Bukreyev A, Collins PL (2008). Newcastle disease virus as a vaccine vector for humans. Curr. Opin. Mol. Ther..

[45] Voysey M (2021). Safety and efficacy of the ChAdOx1 nCoV-19 vaccine (AZD1222) against SARS-CoV-2: an interim analysis of four randomised controlled trials in Brazil, South Africa, and the UK. Lancet.

[46] Sadoff J (2021). Interim results of a phase 1-2a Trial of Ad26.COV2.S Covid-19 Vaccine. N. Engl. J. Med..

[47] Samal, S. K. in *The Biology of Paramyxoviruses* (ed. Samal, S. K.) 69–114 (Caister Academic Press, 2011).

[48] Karron RA (2012). Evaluation of two chimeric bovine-human parainfluenza virus type 3 vaccines in infants and young children. Vaccine.

[49] Bernstein DI (2012). Phase 1 study of the safety and immunogenicity of a live, attenuated respiratory syncytial virus and parainfluenza virus type 3 vaccine in seronegative children. Pediatr. Infect. Dis. J..

[50] Tian JH (2021). SARS-CoV-2 spike glycoprotein vaccine candidate NVX-CoV2373 immunogenicity in baboons and protection in mice. Nat. Commun..

[51] Wrobel AG (2020). SARS-CoV-2 and bat RaTG13 spike glycoprotein structures inform on virus evolution and furin-cleavage effects. Nat. Struct. Mol. Biol..

[52] Bos R (2020). Ad26 vector-based COVID-19 vaccine encoding a prefusion-stabilized SARS-CoV-2 Spike immunogen induces potent humoral and cellular immune responses. NPJ Vaccines.

[53] Amanat, F. et al. Introduction of two prolines and removal of the polybasic cleavage site lead to higher efficacy of a recombinant spike-based SARS-CoV-2 vaccine in the mouse model. *mBio* **12**, 10.1128/mBio.02648-20 (2021). 10.1128/mBio.02648-20 PMC8092267 33653892

[54] Papa G (2021). Furin cleavage of SARS-CoV-2 Spike promotes but is not essential for infection and cell-cell fusion. PLoS Pathog..

[55] Liang B (2016). Packaging and prefusion stabilization separately and additively increase the quantity and quality of respiratory syncytial virus (RSV)-neutralizing antibodies induced by an RSV fusion protein expressed by a parainfluenza virus vector. J. Virol..

[56] Feng L (2020). An adenovirus-vectored COVID-19 vaccine confers protection from SARS-COV-2 challenge in rhesus macaques. Nat. Commun..

[57] Tostanoski LH (2020). Ad26 vaccine protects against SARS-CoV-2 severe clinical disease in hamsters. Nat. Med..

[58] Wu S (2020). A single dose of an adenovirus-vectored vaccine provides protection against SARS-CoV-2 challenge. Nat. Commun..

[59] Corbett KS (2021). mRNA-1273 protects against SARS-CoV-2 beta infection in nonhuman primates. Nat. Immunol..

[60] Riese P, Sakthivel P, Trittel S, Guzman CA (2014). Intranasal formulations: promising strategy to deliver vaccines. Expert Opin. Drug Deliv..

[61] Crowe JE, Collins PL, London WT, Chanock RM, Murphy BR (1993). A comparison in chimpanzees of the immunogenicity and efficacy of live attenuated respiratory syncytial virus (RSV) temperature-sensitive mutant vaccines and vaccinia virus recombinants that express the surface glycoproteins of RSV. Vaccine.

[62] Murphy BR, Sotnikov AV, Lawrence LA, Banks SM, Prince GA (1990). Enhanced pulmonary histopathology is observed in cotton rats immunized with formalin-inactivated respiratory syncytial virus (RSV) or purified F glycoprotein and challenged with RSV 3-6 months after immunization. Vaccine.

[63] Wagner DK (1987). Analysis of immunoglobulin G antibody responses after administration of live and inactivated influenza A vaccine indicates that nasal wash immunoglobulin G is a transudate from serum. J. Clin. Microbiol..

[64] Meyer M (2015). Aerosolized Ebola vaccine protects primates and elicits lung-resident T cell responses. J. Clin. Invest..

[65] Hassan AO (2020). A single-dose intranasal ChAd vaccine protects upper and lower respiratory tracts against SARS-CoV-2. Cell.

[66] Meyer M (2021). Attenuated activation of pulmonary immune cells in mRNA-1273 vaccinated hamsters after SARS-CoV-2 infection. J. Clin. Invest..

[67] Chu H (2020). Comparative tropism, replication kinetics, and cell damage profiling of SARS-CoV-2 and SARS-CoV with implications for clinical manifestations, transmissibility, and laboratory studies of COVID-19: an observational study. Lancet Microbe.

[68] Ren X (2006). Analysis of ACE2 in polarized epithelial cells: surface expression and function as receptor for severe acute respiratory syndrome-associated coronavirus. J. Gen. Virol..

[69] Matsuyama S (2020). Enhanced isolation of SARS-CoV-2 by TMPRSS2-expressing cells. Proc. Natl Acad. Sci. USA.

[70] Kolakofsky D (1998). Paramyxovirus RNA synthesis and the requirement for hexamer genome length: the rule of six revisited. J. Virol..

[71] Subbarao K (2004). Prior infection and passive transfer of neutralizing antibody prevent replication of severe acute respiratory syndrome coronavirus in the respiratory tract of mice. J. Virol..

[72] Clemons DJ, Besch-Williford C, Steffen EK, Riley LK, Moore DH (1992). Evaluation of a subcutaneously implanted chamber for antibody production in rabbits. Lab Anim. Sci..

[73] Walls AC (2020). Structure, function, and antigenicity of the SARS-CoV-2 spike glycoprotein. Cell.

[74] Monaco G (2016). flowAI: automatic and interactive anomaly discerning tools for flow cytometry data. Bioinformatics.

[75] Espitia CM (2010). Duplex real-time reverse transcriptase PCR to determine cytokine mRNA expression in a hamster model of New World cutaneous leishmaniasis. BMC Immunol..

[76] Zivcec M, Safronetz D, Haddock E, Feldmann H, Ebihara H (2011). Validation of assays to monitor immune responses in the Syrian golden hamster (Mesocricetus auratus). J. Immunol. Methods.

[77] Bricker TL (2021). A single intranasal or intramuscular immunization with chimpanzee adenovirus-vectored SARS-CoV-2 vaccine protects against pneumonia in hamsters. Cell Rep..

[78] Wolfel R (2020). Virological assessment of hospitalized patients with COVID-2019. Nature.

[79] Corman, V. M. et al. Detection of 2019 novel coronavirus (2019-nCoV) by real-time RT-PCR. *Euro Surveill* **25**, 10.2807/1560-7917.ES.2020.25.3.2000045 (2020). 10.2807/1560-7917.ES.2020.25.3.2000045 PMC6988269 31992387

[80] Chandrashekar A (2020). SARS-CoV-2 infection protects against rechallenge in rhesus macaques. Science.

