# Supplementary material for: Intranasal immunization with avian paramyxovirus type 3 expressing SARS-CoV-2 spike protein protects hamsters against SARS-CoV-2
Source: NPJ Vaccines. 2022 Jun 28;7:72. doi: 10.1038/s41541-022-00493-x (PMC9240059; doi:10.1038/s41541-022-00493-x)
Supplement: Supplementary file 1 — Supplementary material [file 41541_2022_493_MOESM1_ESM.pdf]

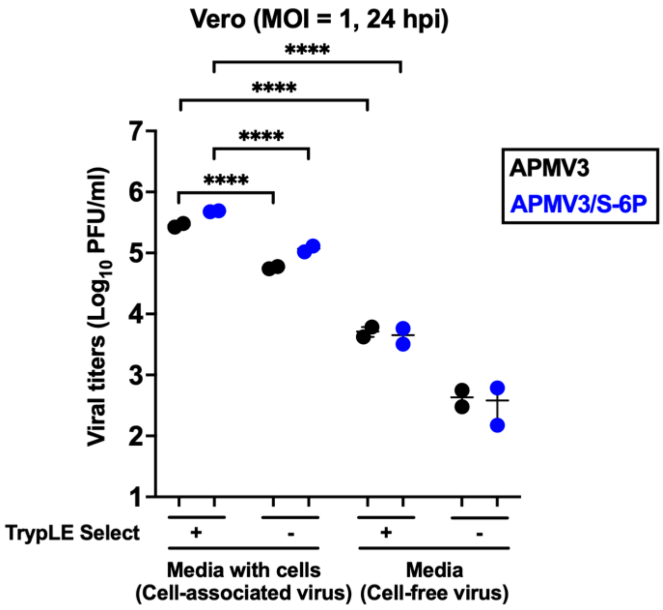

Supplementary figure 1. Effect of exogenous trypsin and harvesting method on APMV3 and APMV3/S-6P yields in Vero cells. Sub-confluent wells of Vero cells in 6-well plates were inoculated in quadruplicate with APMV3 or APMV3/S-6P at an MOI of 1 PFU/cell. After 2 h incubation, Vero cells were washed once with Opti-MEM and replenished with Opti-MEM containing 1% L-glutamine with (+) or without (-) 2% TrypLE Select, as indicated (two wells per condition). To determine the titers of virus released from the infected cells ("Media"), 1 ml of the tissue culture media was harvested at 24 hpi from duplicate wells per virus per condition, clarified by brief centrifugation (3 min at 1,500 rpm), and snap-frozen. To determine the titers of released plus cell-associated virus ("Media with cells"), the monolayers (duplicate wells per virus per condition) were scraped into the 1 ml left of media, and the suspensions were gently vortexed for 1 min and clarified by brief centrifugation (3 min at 1,500 rpm), after which the clarified supernatants were aliquoted and snap-frozen. Virus titers were determined later in duplicate by immunoplaque assay on Vero cells at 37°C as described above. Means with SD from duplicate wells are shown. **** = $p \leq 0.0001$, two-way ANOVA with Tukey post-hoc test.

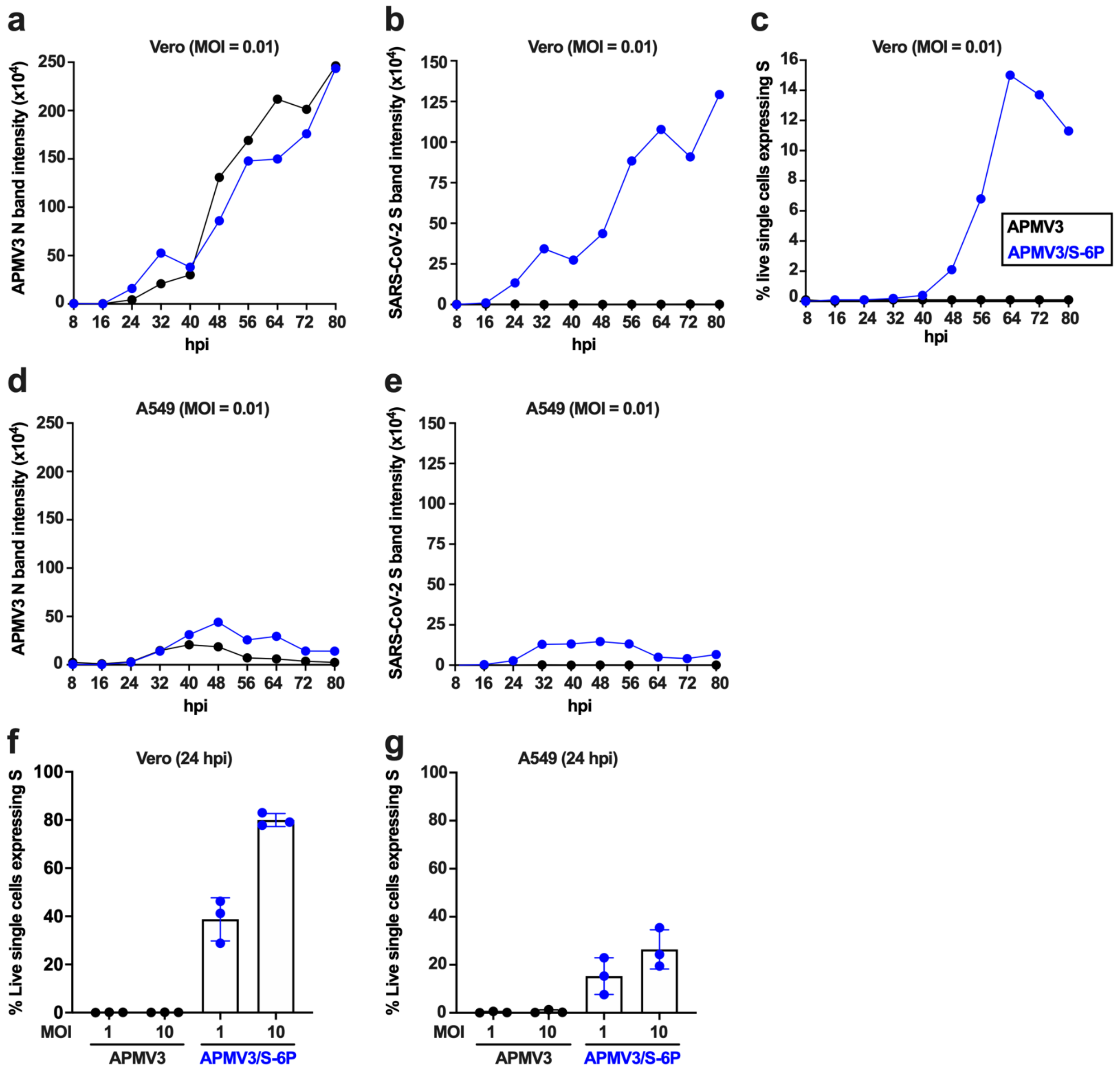

Supplementary figure 2. Expression of virus proteins *in vitro*. Viral protein expression in Vero (a-c) and A549 (d-e) cells. From the time course experiment described in Fig. 1e and 1f, Vero and A549 cells that had been infected in quadruplicate with an MOI of 0.01 PFU/cell of the indicated virus were harvested at 8 h intervals from 8 to 80 hpi. Two wells per time point were harvested for analysis of cell-associated viral proteins by Western blotting (one well) and flow cytometry (one well, Vero cells only). (a, b, d and e) Western blotting time course. The APMV3 N (a, d) and SARS-CoV-2 S (b, e) protein bands were visualized by Western blotting using a primary rabbit anti-APMV3 serum and a primary goat anti-SARS-CoV-2 S serum, respectively, and labeled with infrared dye-conjugated secondary antibodies (see Materials and Methods). The level of expression of each protein band was normalized to β-actin blotted from a replicate gel lane of the same sample, and expressed as fluorescence intensity. (c) Flow cytometry time course. The percentage of live single SARS-CoV-2 S-expressing cells in APMV3/S-6P-infected Vero cells was determined by flow cytometry using a PE-conjugated CR3022 anti-SARS-CoV-2 S human monoclonal antibody. (f, g) In an additional set of

three independent experiments, Vero (f) and A549 (g) cells were infected with the indicated virus using an MOI of 1 or 10 PFU/cell. At 24 hpi, cells were harvested and stained using the PE-conjugated CR3022 anti-SARS-CoV-2 S monoclonal antibody, as described above. Cells were analyzed by flow cytometry and the % of live single cells expressing SARS-CoV-2 S was determined. The mean and SD are shown.

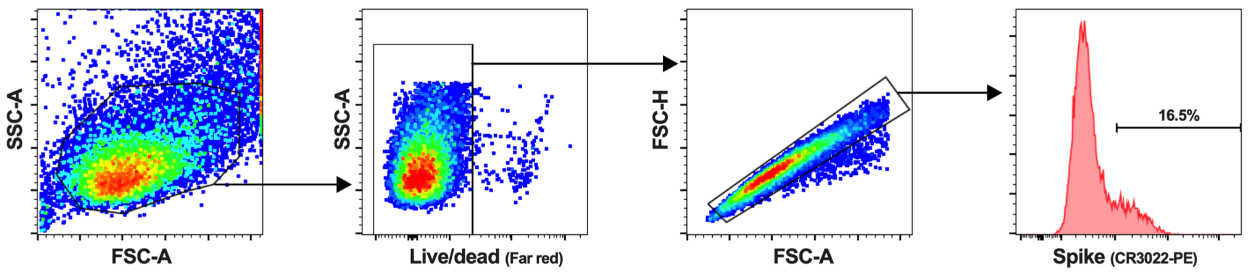

Supplementary figure 3. Gating strategy to identify SARS-CoV-2 spike-expressing cells described in supplementary figure 2c, f and g. Representative flow cytometry dot plots and histogram of A549 cells describing the typical gating strategy used to identify SARS-CoV-2 spike-expressing cells. Forward and size scatter areas were first used to identify intact cells. Live cells were next gated based on a live/dead staining. Then, singlets were selected using a gate based on forward scatter height and forward scatter area. The SARS-CoV-2 S-expressing cells were identified using a PE-conjugated human CR3022 mAb and expressed as a percentage of live single cells.

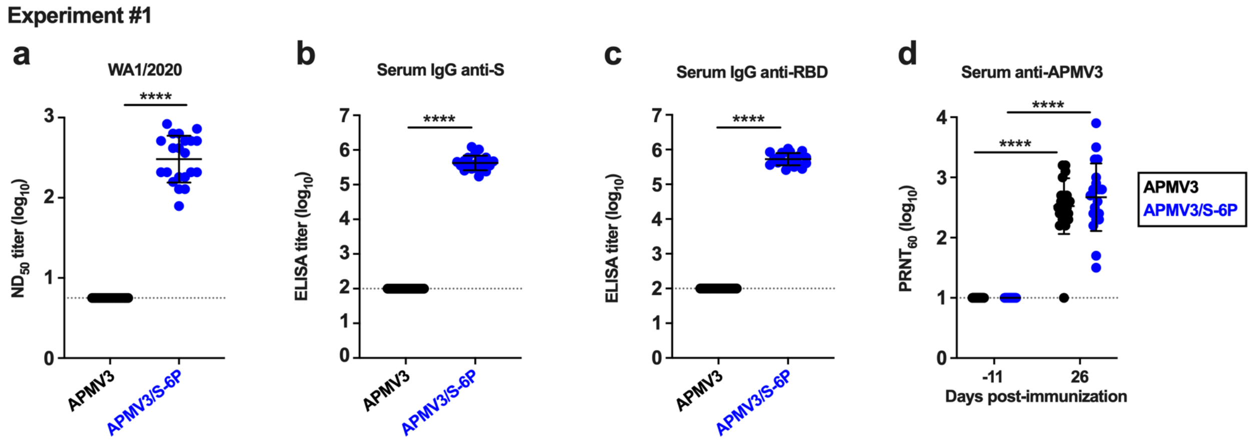

Supplementary figure 4. Immunogenicity of APMV3 and APMV3/S-6P in hamsters (from Experiment #1). From Experiment #1 (as shown in the timeline in Fig. 3a) sera were collected on day 26 pi from 21 hamsters per group that had been inoculated on day 0 with 6 log_{10} PFU of APMV3/S-6P or APMV3. The sera were analyzed as follows: (a) ND_{50} titers expressed in log_{10} against SARS-CoV-2 WA1/2020, evaluated on Vero E6 cells. The limit of detection, indicated by a dotted line, is 0.75 log_{10}. (b and c) End-point ELISA titers expressed in log_{10} of serum IgG to the full-length S (b) and the RBD of S (c). The limit of detection, indicated by a dotted line, is 2 log_{10}. (d) Titers of APMV3-neutralizing antibodies in sera from day -11 and 26, expressed in log_{10} 60% plaque reduction neutralization titer ($PRNT_{60}$). The limit of detection is indicated by a dotted line (1 log_{10}). In each graph, the mean and SD are shown. **** = $p \leq 0.0001$, Mann-Whitney test (a-c) or two-way ANOVA with Sidak post-hoc test (d).

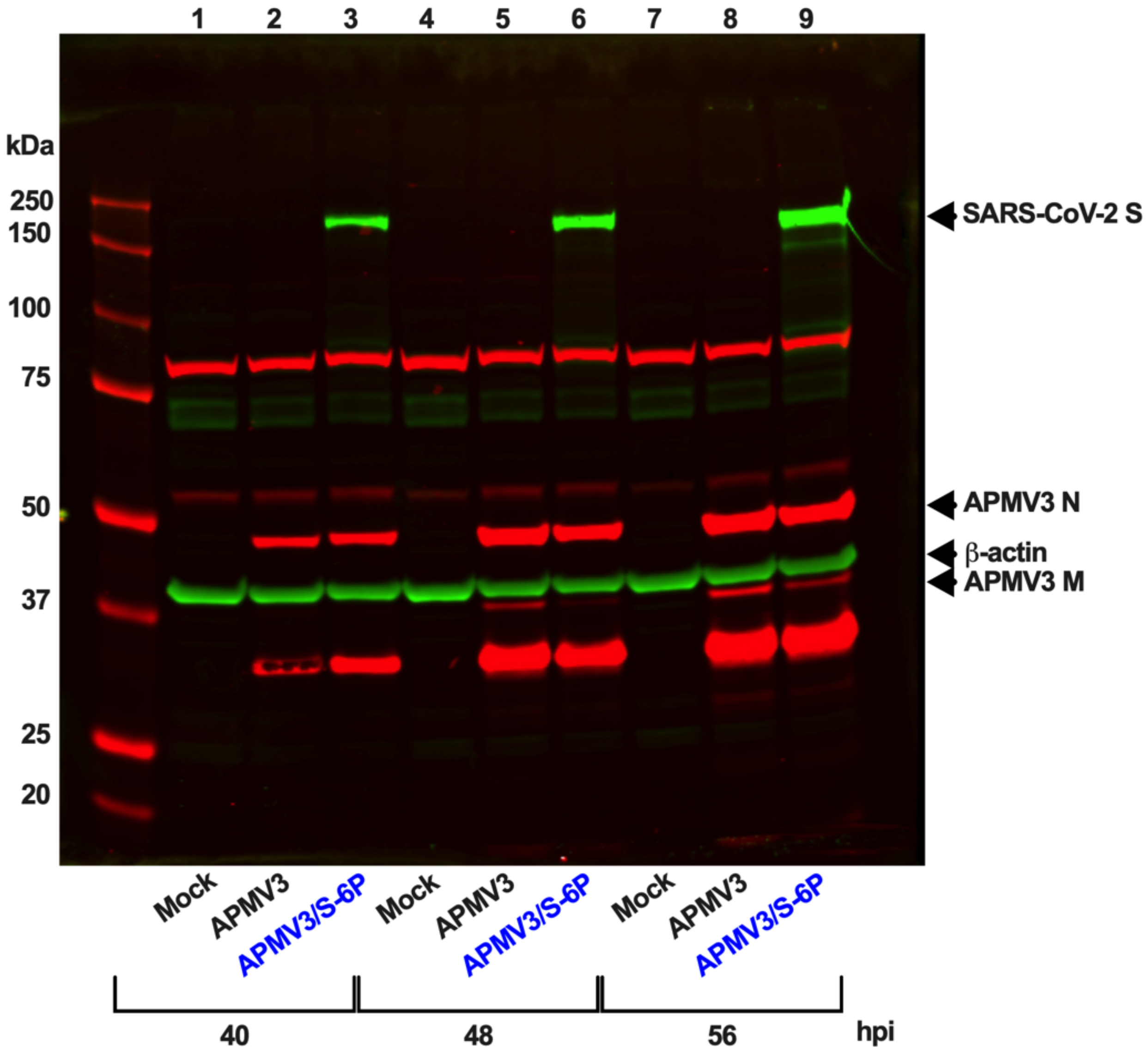

Supplementary figure 5. Uncropped and unprocessed scan that was used to generate Fig. 2a, showing viral protein expression in Vero cells. From the experiment described in Fig. 1e and 1f, one additional replicate well of Vero cells per virus, which had been infected with an MOI of 0.01 PFU/cell of the indicated virus, was harvested at 40, 48, and 56 hpi, and cell lysates were prepared and analyzed by SDS-PAGE under reducing and denaturing conditions. The APMV3 N and SARS-CoV-2 S protein bands were visualized by Western blotting using primary rabbit anti-APMV3 serum and goat anti-SARS-CoV-2 S serum, followed by incubation with IRDye-conjugated secondary antibodies and infrared imaging (see Materials and Methods). A mouse anti-β-actin antibody was included to provide a loading control. Panels were derived from the same gel. Lanes 4-6 (cells harvested at 48 hpi) were used to generate Fig. 2a.
