# Supplementary material for: Intranasal immunization with avian paramyxovirus type 3 expressing SARS-CoV-2 spike protein protects hamsters against SARS-CoV-2
Source: NPJ Vaccines. 2022 Jun 28;7:72. doi: 10.1038/s41541-022-00493-x (PMC9240059; doi:10.1038/s41541-022-00493-x)
Supplement: Supplementary file 2 — Reporting Summary [file 41541_2022_493_MOESM2_ESM.pdf]

nature portfolio

Corresponding author(s): Ursula J Buchholz, Cyril Le Nouen

Last updated by author(s): Mar 4, 2022

Reporting Summary

Nature Portfolio wishes to improve the reproducibility of the work that we publish. This form provides structure for consistency and transparency in reporting. For further information on Nature Portfolio policies, see our Editorial Policies and the Editorial Policy Checklist.

Statistics

For all statistical analyses, confirm that the following items are present in the figure legend, table legend, main text, or Methods section.

n/a	Confirmed	
☐	☒	The exact sample size (*n*) for each experimental group/condition, given as a discrete number and unit of measurement
☐	☒	A statement on whether measurements were taken from distinct samples or whether the same sample was measured repeatedly
☐	☒	The statistical test(s) used AND whether they are one- or two-sided<br>*Only common tests should be described solely by name; describe more complex techniques in the Methods section.*
☒	☐	A description of all covariates tested
☐	☒	A description of any assumptions or corrections, such as tests of normality and adjustment for multiple comparisons
☐	☒	A full description of the statistical parameters including central tendency (e.g. means) or other basic estimates (e.g. regression coefficient) AND variation (e.g. standard deviation) or associated estimates of uncertainty (e.g. confidence intervals)
☐	☒	For null hypothesis testing, the test statistic (e.g. *F*, *t*, *r*) with confidence intervals, effect sizes, degrees of freedom and *P* value noted<br>*Give P values as exact values whenever suitable.*
☒	☐	For Bayesian analysis, information on the choice of priors and Markov chain Monte Carlo settings
☒	☐	For hierarchical and complex designs, identification of the appropriate level for tests and full reporting of outcomes
☒	☐	Estimates of effect sizes (e.g. Cohen's *d*, Pearson's *r*), indicating how they were calculated

Our web collection on statistics for biologists contains articles on many of the points above.

Software and code

Policy information about availability of computer code

Data collection	BD Facs Diva<br>ImageStudio 5.2<br>QuantStudio 6/7 Pro Touchscreen instrument Operating Software<br>BioTek Gen5
Data analysis	Flowjo version 10.8<br>ImageStudioLite 5.2.5<br>Genesis 1.8.1<br>GraphPad Prism version 9<br>Relative Quantification, Standard Curve on https://apps.thermofisher.com

For manuscripts utilizing custom algorithms or software that are central to the research but not yet described in published literature, software must be made available to editors and reviewers. We strongly encourage code deposition in a community repository (e.g. GitHub). See the Nature Portfolio guidelines for submitting code & software for further information.

Data

Policy information about availability of data

All manuscripts must include a data availability statement. This statement should provide the following information, where applicable:

- Accession codes, unique identifiers, or web links for publicly available datasets
- A description of any restrictions on data availability
- For clinical datasets or third party data, please ensure that the statement adheres to our policy

The datasets generated and/or analyzed during the current study are available within the main and supplemental figures. All data is available from the corresponding authors .

Field-specific reporting

Please select the one below that is the best fit for your research. If you are not sure, read the appropriate sections before making your selection.

☒ Life sciences ☐ Behavioural & social sciences ☐ Ecological, evolutionary & environmental sciences

For a reference copy of the document with all sections, see nature.com/documents/nr-reporting-summary-flat.pdf

Life sciences study design

All studies must disclose on these points even when the disclosure is negative.

Sample size	In hamster experiment #1, animals were divided in 2 treatment groups of 45 animals each. The study included 3 time points to evaluate vaccine replication; in each group, n=6 per time point were used to evaluate vaccine replication, and n=2 per time point were used for immunohistochemistry analyses. The remaining 21 animals per treatment group were used for serology analyses. In experiment #2, hamsters were divided in 2 treatment groups of 10 animals each. Subsets of n=5 per time point were used to evaluate protection against SARS-CoV-2 challenge.
Data exclusions	No data were excluded.
Replication	Two separate hamster experiments were performed. The level of immunogenicity induced by the APMV3/S-6P vaccine candidate in the two animal experiments was comparable.
Randomization	Hamsters were randomly assigned to experimental groups.
Blinding	While formal blinding was not performed in this study, animal technicians involved in weighing and clinical observations remained blinded, and analyses of animal study samples were performed in a blinded manner whenever possible. Statistics were not calculated until the study was complete and all data had been verified to be accurate.

Reporting for specific materials, systems and methods

We require information from authors about some types of materials, experimental systems and methods used in many studies. Here, indicate whether each material, system or method listed is relevant to your study. If you are not sure if a list item applies to your research, read the appropriate section before selecting a response.

Materials & experimental systems

n/a	Involved in the study	
☐	☒	Antibodies
☐	☒	Eukaryotic cell lines
☒	☐	Palaeontology and archaeology
☐	☒	Animals and other organisms
☒	☐	Human research participants
☒	☐	Clinical data
☒	☐	Dual use research of concern

Methods

n/a	Involved in the study	
☒	☐	ChIP-seq
☐	☒	Flow cytometry
☒	☐	MRI-based neuroimaging

Antibodies

Antibodies used	Included in the methods section of the manuscript.
Validation	Chicken APMV3 antiserum was provided by Dr. Siba Samal, University of Maryland and was validated in-house by Western blot and immunoplaque assay.<br>Rabbit APMV3 antiserum was produced using sucrose-purified recombinant APMV3. Prior to use, the specificity of the hyperimmune

serum was validated in-house by Western blot, plaque-reduction neutralization assay, and immunoplaque assay.
Goat hyperimmune serum to the SARS-CoV-2 spike protein was produced and validated in-house by Western blot, SARS-CoV-2 neutralization assays (BSL3), and immunoplaque assay. The hyperimmune serum detects recombinantly-expressed SARS-CoV-2 S protein at the expected size in Western blots, and efficiently neutralizes live SARS-CoV-2 in virus neutralization assays performed in BSL3 on Vero E6 cells (ND50 to USA/WA1/2020: 11.0 log2).
Anti-S-RBD human CR3022 antibody was produced and validated in-house by immunoplaque assay.
All secondary antibodies are commercially available and validated by the manufacturers.

Eukaryotic cell lines

Policy information about cell lines

Cell line source(s)	A549 and Vero E6 cells were from ATCC. Vero cells were originally from ATCC, amplified under GMP by CRL.<br>The BSR T7/5 cell line was previously described (Buchholz UJ et al., J Virol 1999).<br>The generation of Vero E6 cells stably expressing TMPRSS2 was described previously (Liu X et al., PNAS 2021).
Authentication	Each ATCC cell line was provided with a certificate of analysis. The cell identity was verified by morphology. Vero cells were originally from ATCC. Their karyotype was confirmed by CRL.
Mycoplasma contamination	All cell lines were tested to be negative for mycoplasma.
Commonly misidentified lines<br>(See ICLAC register)	n/a

Animals and other organisms

Policy information about studies involving animals; ARRIVE guidelines recommended for reporting animal research

Laboratory animals	Golden Syrian hamsters, 5 to 6 week old, Envigo Laboratories.
Wild animals	No wild animals were used in this study.
Field-collected samples	The study did not involve samples collected from the field.
Ethics oversight	The hamster studies were approved by the NIAID Animal Care and Use Committee. All the animal experiments were carried out following the Guide for the Care and Use of Laboratory Animals by the NIH.

Note that full information on the approval of the study protocol must also be provided in the manuscript.

Flow Cytometry

Plots

Confirm that:

- ☐ The axis labels state the marker and fluorochrome used (e.g. CD4-FITC).
- ☐ The axis scales are clearly visible. Include numbers along axes only for bottom left plot of group (a 'group' is an analysis of identical markers).
- ☐ All plots are contour plots with outliers or pseudocolor plots.
- ☒ A numerical value for number of cells or percentage (with statistics) is provided.

Methodology

Sample preparation	See methods section: Mock- or virus-infected Vero or A549 cells in 6-well plates were trypsinized with TrypLE Select and harvested at the indicated time points. Next, cells were stained with LIVE/DEAD Fixable Near-IR Dead Cell Stain Kit (Thermo Fisher Scientific) and fixed with Cytofix/Cytoperm (BD Biosciences). After permeabilization in Perm/Wash buffer (BD Biosciences), cells were incubated with a phycoerythrin (PE)-conjugated human anti-SARS-CoV-2 S monoclonal antibody (CR3022) (1:100) in Perm/Wash buffer for 20 min in the dark. Stained cells were washed five times with Perm/Wash buffer and resuspended in PBS until analyzed by a FACSymphony Flow Cytometer (BD Biosciences).
Instrument	FACS Symphony Flow cytometer (BD Biosciences)
Software	Flowjo software version 10
Cell population abundance	Flow cytometry experiments were performed using ATCC-characterized Vero or A549 cell lines. At least 20,000 events were acquired for each sample.

Gating strategy

Quality control of each acquired sample was performed using the FlowAI plugin on FlowJo. Then, compensation was performed automatically using beads for each antibody. Live/dead staining, forward scatter height, and forward scatter area were used to identify single live cells. The expression of the SARS-CoV-2 S protein was analyzed on single live cells using uninfected cells or cells infected with the empty vector as controls.

☒ Tick this box to confirm that a figure exemplifying the gating strategy is provided in the Supplementary Information.
